# Immunological Fingerprints of Controllers Developing Neutralizing HIV-1 Antibodies

**DOI:** 10.1016/j.celrep.2019.12.087

**Published:** 2020-01-28

**Authors:** Enrique Martin-Gayo, Ce Gao, Hsiao Rong Chen, Zhengyu Ouyang, Dhohyung Kim, Kellie E. Kolb, Alex K. Shalek, Bruce D. Walker, Mathias Lichterfeld, Xu G. Yu

**Affiliations:** 1 Ragon Institute of MGH, MIT, and Harvard, Boston, MA, USA; 2 Universidad Autónoma de Madrid, Hospital de la Princesa, Madrid, Spain; 3 Department of Chemistry, MIT Institute for Medical Engineering and Science (IMES), MIT, Cambridge, MA, USA; 4 Koch Institute for Integrative Cancer Research, MIT, Cambridge, MA, USA; 5 Broad Institute of MIT and Harvard, Cambridge, MA, USA; 6 Howard Hughes Medical Institute, Chevy Chase, MD, USA; 7 Infectious Disease Division, Brigham and Women’s Hospital, Boston, MA, USA; 8 Infectious Disease Division, Massachusetts General Hospital, Boston, MA, USA

**Keywords:** HIV, dendritic cell, systems biology, CD4 T cell, bnAb, controller, neutralizer, monocyte, B cell, RNAseq

## Abstract

The induction of broadly neutralizing antibodies (bnAbs) is highly desired for an effective vaccine against HIV-1. Typically, bnAbs develop in patients with high viremia, but they can also evolve in some untreated HIV-1 controllers with low viral loads. Here, we identify a subgroup of neutralizer-controllers characterized by myeloid DCs (mDCs) with a distinct inflammatory signature and a superior ability to prime T follicular helper (Tfh)-like cells in an STAT4-dependent fashion. This distinct immune profile is associated with a higher frequency of Tfh-like cells in peripheral blood (pTfh) and an enrichment for Tfh-defining genes in circulating CD4^+^ T cells. Correspondingly, monocytes from this neutralizer controller subgroup upregulate genes encoding for chemotaxis and inflammation, and they secrete high levels of IL-12 in response to TLR stimulation. Our results suggest the existence of multi-compartment immune networks between mDCs, Tfh, and monocytes that may facilitate the development of bnAbs in a subgroup of HIV-1 controllers.

## Highlights

• HIV-1 controllers with neutralizing Abs are subdivided in two subgroups (Nt1 and Nt2)
• HIV-1-specific antibodies from Nt2 individuals display superior neutralization potency
• Nt2 exhibit distinct transcriptional signatures in DC, monocytes, and CD4 T cells
• Transcriptional and functional data suggest improved DC-pTFH interactions in Nt2

## Graphical Abstract

**Figure:**
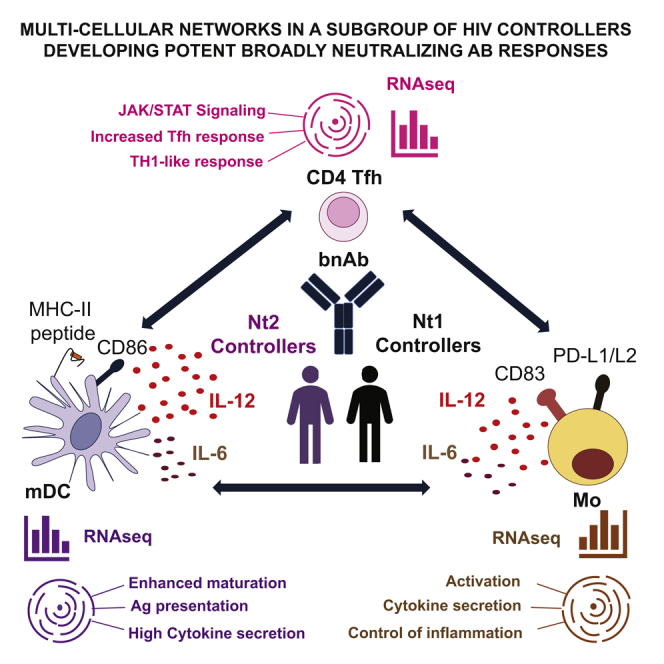

## Teaser

Martin-Gayo et al. identify a subgroup of HIV-1 controllers who mount potent neutralizing antibodies against the virus. The distinguishing features of this subset of individuals include a tightly regulated network of transcriptional and functional interactions between dendritic cells, T cells, and monocytes.

## Introduction

The induction of antibodies (Abs) with broad neutralizing activity against different HIV-1 strains (bnAbs) is a promising strategy for the development of protective and therapeutic vaccines. To date, the exact mechanisms enabling the evolution of bnAbs remain unclear, despite previous efforts to explore the potential involvement of individual cell types in the induction of bnAbs. For example, CXCR5^+^ PD-1^+^ T follicular helper CD4^+^ cells (Tfh) play a critical role in supporting humoral immune responses due to their ability to promote B cell growth and proliferation, immunoglobulin (Ig) class switching, and somatic hypermutation, and are statistically associated with the development of bnAbs in HIV-1-infected patients (Martin-Gayo et al., 2017, Locci et al., 2013). Myeloid dendritic cells (mDCs) and monocytes (Mos) contribute to humoral immune responses through the direct stimulation of B cell maturation and survival (Litinskiy et al., 2002, Ueno et al., 2010) and indirectly by facilitating naive CD4^+^ T cells differentiation into CXCR5^+^ PD-1^+^ Tfh cells in the presence of B cells (Martin-Gayo et al., 2017).

Current concepts imply that the development of bnAbs requires prolonged exposure to viral antigen (Ag), which preferentially occurs in HIV-1 patients with high levels of plasma viremia and elevated immune inflammation, conditions that do not adequately reflect the immune environments in possible vaccine recipients. However, a small proportion (30%) of HIV-1 controllers is capable of developing HIV-1-specific Abs with broader neutralizing activity under low plasma viremia conditions (neutralizers [Nts]) and represent a more suitable model to study the mechanisms required for effective humoral responses against HIV-1 for vaccination purposes (Doria-Rose et al., 2010, Chen et al., 2011, Migueles et al., 2002, Sáez-Cirión and Pancino, 2013, Ranasinghe et al., 2015). The initial studies in this patient population suggested that inflammatory cytokine profiles (Dugast et al., 2017), a relative enrichment in circulating, long-lived PD-1^Lo^ memory Tfh precursors, and an improved ability of mDCs to prime such Tfh precursors favor the development of increased Ab neutralization breadth (Martin-Gayo et al., 2017). However, HIV-1 controllers represent a heterogeneous population of individuals in whom histocompatibility leukocyte antigen (HLA)-restricted CD8^+^ and CD4^+^ T cell responses (Walker and Yu, 2013, Vigneault et al., 2011, Ranasinghe et al., 2015), innate immune modulation, and Ag recognition (Martin-Gayo et al., 2015) or Ab responses may play different or complementary roles in HIV-1 immune control.

In the present study, we hypothesized that the development of bnAbs in HIV-1 controllers is the result of reciprocal cell interactions leading to distinct immune circuits between B cells, mDCs, Mos, and Tfh. To analyze this, we studied transcriptional profiles in primary mDCs from Nt and non-neutralizer (NN) controllers and their associations with gene expression signatures of CD4^+^ T cells, Mos, and B cells using RNA sequencing (RNA-seq). Our unbiased analytical approach identified a subgroup of Nt controllers defined by a distinct transcriptional pattern of mDCs, CD4^+^T cells, and Mos, coupled with a higher Ag-presenting cell function of mDCs to induce Tfh cells *in vitro*.

## Results

### Transcriptional Profiles of mDCs Identify a Subgroup of Nt Controllers with Potent Neutralizing Abs against HIV-1

**Figure 1:**
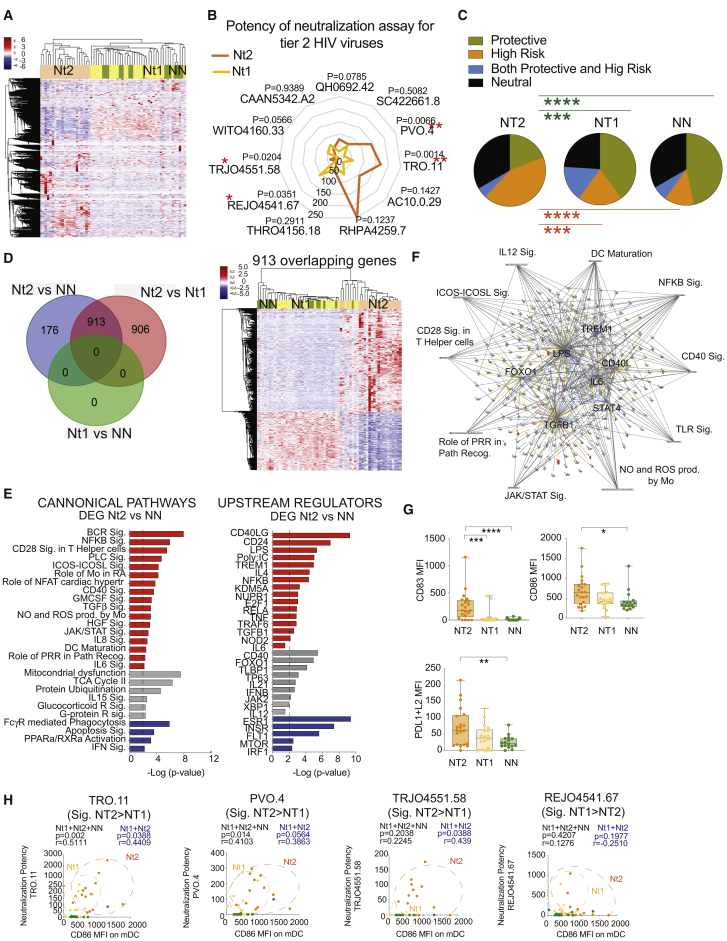
Transcriptional Signatures of mDCs Identify a Subgroup of Nt Controllers with Potent Neutralizing Ab Responses (A) Heatmap showing unsupervised hierarchical clustering of non-neutralizer (NN) and neutralizer (Nt) controller patients based on the transcriptional expression patterns of 13,239 genes in mDCs. (B) Spider diagram showing the potency of neutralization of 11 tier 2 and tier 3 viruses by plasma neutralizing Abs from Nt1 (yellow) and Nt2 (orange) controllers. ^∗∗^p < 0.01, Mann-Whitney *U* test. (C) Pie charts representing the proportions of protective (green), high-risk (orange), both (blue) or neither protective or high-risk (black) HLA class I B alleles (see Table S4). ^∗∗∗^p < 0.001, ^∗∗∗∗^p < 0.0001, chi-square test. (D) Left panel shows Venn diagram illustrating the overlap of differentially expressed genes (DEGs) in mDCs from the indicated study groups using an FDR-adjusted p < 10e−5. Right panel shows heatmap representing unsupervised hierarchical cluster distribution of Nt2 (orange), Nt1 (yellow), and NN (green) based on the expression of 913 overlapping DEGs between DC from Nt2 versus Nt1 and from Nt2 versus NN. (E) Selected significant canonical pathways (left) and upstream regulators (right) predicted by Ingenuity Pathway Analysis (IPA) from DEGs between mDCs from Nt2 and NN controllers. Predicted upregulated and downregulated pathways and regulators are highlighted in red and blue, respectively. Gray highlights pathways and regulators for which no directional change can be determined. Significance cutoff was established at −log p value = 2. (F) Network analysis of selected upstream regulators (highlighted inside) and canonical pathways (highlighted outside) among DEGs between mDCs from Nt2 compared to NN controllers. Significance cutoff was established at −log p value = 2. (G) Box and whiskers plot showing mean fluorescence intensity (MFI) of surface CD83 (upper left) and CD86 (lower right) and PDL1+L2 (lower left) expression in mDCs from Nt2 (n = 21), Nt1 (n = 18), and NN (n = 16) controllers. The error bars represent minimum to maximum values. Statistical significance was calculated using a two-tailed Mann-Whitney test. ^∗^p < 0.05, ^∗∗∗^p < 0.001, ^∗∗∗∗^p < 0.0001. (H) Spearman correlation between the MFI of CD86 on mDCs and corresponding potency of antibody neutralization of the indicated tier 2 HIV-1 pseudoviruses differentially neutralized by plasma from Nt2 versus Nt1 patients. FDR-corrected p and R values of combined Nt1 (yellow) and Nt2 (orange) or all patient cohorts, including NNs (green), are indicated in blue and black, respectively.

To evaluate the potential association of mDCs with the natural evolution of bnAbs in HIV-1 controllers in detail, we sorted circulating Lin^−^ CD14^−^ CD11c^+^ HLA-DR^+^ mDCs from the blood of antiretroviral therapy (ART)-naive controllers with low plasma HIV-1 viral loads (VLs; <2,000 copies/mL, except for 7 outliers with a single viral blip) and with (Nts, n = 45) or without (NNs, n = 15) detectable Abs capable of neutralizing a panel of 11 tier 2 HIV-1 pseudoviruses (Figure S1). These controller subgroups did not significantly differ in regard to CD4^+^ T cell counts or infection duration, but Nts were characterized by significantly higher VLs, despite overall low viremia (Figures S1A and S1B). Subsequently, transcriptional profiles of mDCs from these cohorts were analyzed by RNA-seq. Unsupervised clustering based on the expression of 13,239 genes in mDCs revealed that the gene signatures of a portion of Nts (Nt1) overlapped with those observed in NNs, while a separate subgroup of Nts (Nt2) displayed a discrete mDCs gene expression profile relative to the other patient groups (Figure 1A). Notably, Nt1 and Nt2 controllers did not differ significantly in terms of viral loads, CD4^+^ T cells, duration of HIV-1 infection, or neutralizing Ab breadth, although Nt2 individuals tended to display a higher potency of neutralization than Nt1 controllers for 6 of the 11 tier 2 HIV-1 pseudoviruses tested (Figures 1B, S1B, and S1C; Table S1). The identification of Nt2 patients was not driven by the differences in sample collection time between Nts and NNs (Figure S2A). Nt2 controllers were significantly enriched for HLA-B alleles associated with rapid disease progression, as opposed to Nt1 Nt and NN controllers, who exhibited elevated frequencies of protective HLA-B alleles linked to the natural control of HIV-1 replication (Bashirova et al., 2014) (Figure 1C; Table S4).

We next focused on understanding the transcriptional signatures of mDCs from Nt2 patients. While there was a low statistical difference in gene expression patterns between NN and Nt1 patients, we observed n = 1,089 and n = 1,819 differentially expressed genes (DEGs; false discovery rate [FDR]-corrected p < 10^−5^) when comparing the transcriptional patterns of mDCs from Nt2 to those from NN and Nt1 controllers, respectively (Figure 1D). Notably, 913 genes from these 2 different sets of DEGs overlapped with one another and allowed us to distinguish Nt2 controllers from the other 2 patient subgroups by unsupervised clustering. Subsequent Ingenuity Pathway Analysis (IPA) of DEGs expressed in mDCs from Nt2 versus NN controllers revealed enrichment of Nt2 mDCs, with transcripts related to T cell co-stimulation (CD40, CD28, ICOS), improved B cell receptor signaling, and activation of cytokine signaling (Figures 1E, S2B, and S2C), suggesting an enhanced functional state of mDCs from Nt2 controllers compared to Nt1 and NN individuals. Similar results were observed when we analyzed the pathways predicted for the 913 overlapping DEGs between mDCs from Nt2 and Nt1 (Figure S3D) or the non-overlapping DEGs from Nt2 versus Nt1 signatures (Figures S3A–S3D). Genes correlated with Ab breadth (Figures S3E–S3H) were also predicted to be related to B cell maturation and cellular activation and maturation. Consistent with this finding, upstream regulators predicted to govern the transcriptional signature of mDCs from Nt2 included activating Toll-like receptor (TLR) ligands and immunomodulatory cytokines known to induce the functional maturation of mDCs (Figures 1E and 1F). Moreover, a phenotypical analysis of circulating mDCs from our 3 controller subgroups (Figures 1G and S3I) indicated that cells from Nt2 expressed significantly higher levels of co-stimulatory molecules such as CD83, CD86, PD-L1, PD-L2, and CD40 than NNs or Nt1 Nt controllers. The higher expression levels of CD86 on mDCs were specifically correlated with the higher potency of neutralization for 3 of 4 HIV-1 pseudoviruses that were more efficiently neutralized by plasma from Nt2 patients (Figures 1B–1H and S1C); less significant trends were observed for CD83 and PDL1+L2 expression and viral neutralization (Figure S4). The higher expression of CD86 and CD83 on mDCs was not correlated with plasma VLs, although some association was found for PD-L1+L2 and CD83 within the Nt2 patients (Figure S2D). Our data identify a subset of Nt controllers, called Nt2 in this article, that is characterized by mDCs with transcriptional and phenotypic signs of enhanced functional activity, coupled with a superior potency of neutralizing Ab responses against HIV-1.

### Interconnected, Multi-compartment Immune Signatures in Nt2 Patients

**Figure 2:**
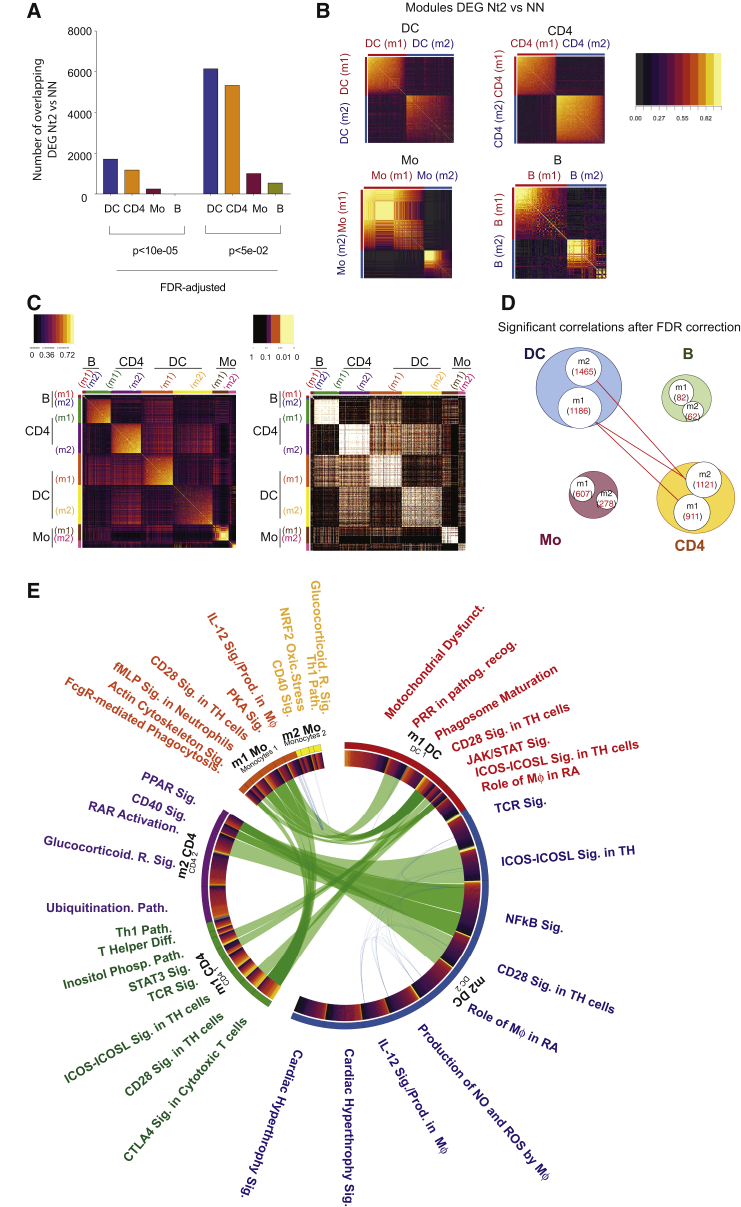
Immunological Networks between mDCs and CD4^+^ T Cells in Nt2 Controller (A) Numbers of DEGs in mDCs (blue), CD4^+^ T cells (orange), Mos (purple), and B cells (green) from Nt2 controllers compared to NNs (FDR-adjusted p < 10e−5. (B) Heatmaps reflecting modules (m) of genes with correlated expression intensity among DEGs from Nt2 versus NN in mDCs, CD4^+^T cells, Mos, and B cells. (C) Heatmaps reflecting correlation coefficients (left panel) and corresponding FDR-corrected significance levels determined by a whole-gene correlation network analysis (WGCNA) between indicated gene modules identified in (B). (D) Schematic representation of gene modules identified in (B) that are significantly (FDR-adjusted p < 0.05) associated between indicated cell populations. Numbers indicate numerical counts of genes within each gene module. (E) Circos plot representing interactions among pathways predicted by IPA among gene modules m1 and m2 from mDCs, CD4^+^ T cells, and Mos. Connections were defined by >50% of genes with correlated gene expression between 2 pathways (p < 0.05, Spearman correlation). Connecting blue lines represent individual DEGs between Nt2 versus NN across different cell types. Thick connecting green lines represent transcriptional pathways indicated on the external labeling.

Given that the evolution of bnAbs represents a multifactorial process, we hypothesized that distinct transcriptional signatures in mDCs from Nt2 individuals compared to NNs may be part of the immune circuits involving additional immune cell types. To address this, we analyzed RNA-seq data from sorted CD4^+^ T cells, B cells, and Mos from the Nt2, Nt1, and NN controllers. As shown in Figure 2A, mDCs and CD4^+^ T cells from Nt2 expressed the largest number of DEGs compared to NN patients, and DEGs from each of these populations exhibited two modules with tightly correlated transcriptional activity (Figure 2B). Transcriptional differences among the patient populations were less pronounced in peripheral blood Mo and B cells, and clusters of correlated gene expression patterns and correlation coefficients among DEGs were weaker for Mos and B cells (Figure 2B). To further evaluate the associations between the transcriptional signatures of mDCs and the other immune cell types in Nt2 patients, we performed a whole-gene correlation network analysis (WGCNA) (Langfelder and Horvath, 2008) between the modules of DEGs previously defined for each cell type (Figure 2B) in Nt2 controllers. As shown in Figures 2C and 2D, gene modules 1 and 2 from mDCs showed a high-level positive association (p > 0.05 after FDR correction; Figure 2D; Table S2) with modules 1 and 2 from CD4^+^ T cells. In contrast, correlations between modules 1 and 2 from Mos and module 2 from CD4^+^ T cells were less obvious and failed to reach strong statistical significance after FDR correction. A functional computational analysis of each gene module indicated significant reciprocal associations between immune recognition and immune activation pathways in mDCs, CD4^+^ T cells, and Mos from Nt2 patients, indicating close connections between individual functional modules from these different cell compartments (Figure 2E). These results suggest that Nt2 controllers are characterized by interrelated, multi-compartment immune cell meta-signatures involving mostly mDCs, CD4^+^ T cells, and Mos.

### Functional Interactions between mDCs and CD4^+^ T Cells in Nt2 Patients

**Figure 3:**
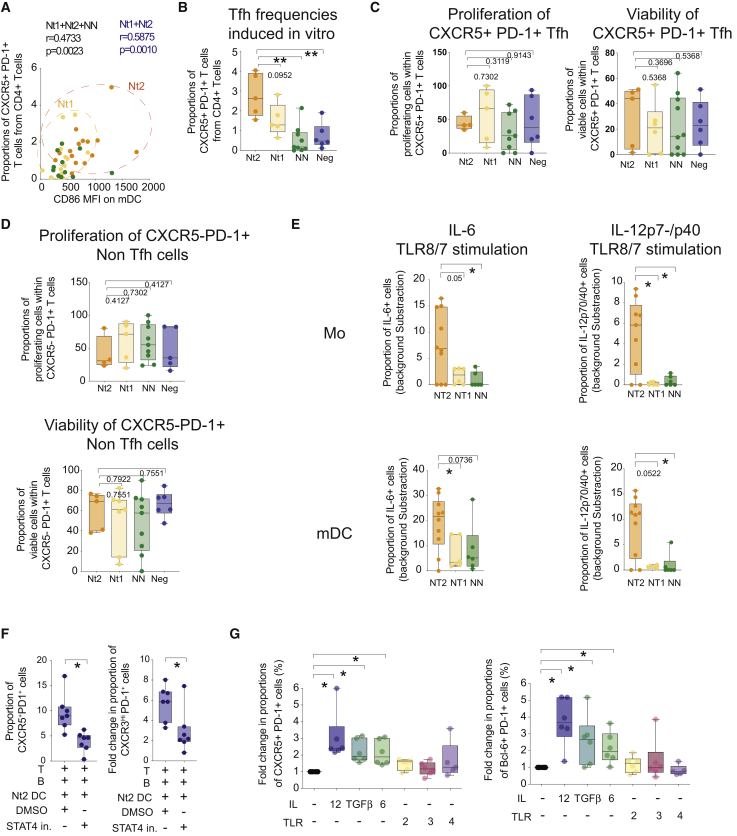
IL-12 Signaling Is Required for Superior Tfh-Priming Properties of mDCs from Nt2 Controllers (A) Spearman correlation between frequencies of CXCR5^+^ PD-1^+^ pTfh from total CD4^+^ T cells and MFI of CD86 in mDCs from indicated controller Nts. Nt1 and Nt2 patients are highlighted in yellow and orange, respectively. R and p values for all Nts or all controllers are indicated in blue and black, respectively. (B) Box and whiskers plot showing the proportions of CXCR5^+^ PD-1^+^ Tfh-like cells induced from naive CD4^+^ T cells after 6 days of culture in the presence of autologous naive B cells and allogeneic mDCs from either Nt2 (orange, n = 5) or Nt1 (yellow, n = 5) Nts, NN (green, n = 9) controllers, and healthy donors (blue, n = 6). The error bars represent minimum to maximum values. Statistical significance was calculated using a two-tailed Mann-Whitney test. ^∗∗^p < 0.01. (C) Box and whiskers plot showing the proportions of proliferating carboxyfluorescein succinimidyl ester low (CFSE low) (left panel) and viable (right panel) cells within CXCR5^+^ PD-1^+^ Tfh-like cells present in culture after incubation with mDCs from Nt2 (orange, n = 4 and 5) and Nt1 (yellow, n = 5 and 6) Nts, NNs (green, n = 9 and 10), and healthy donors (blue, n = 6). The error bars represent minimum to maximum values. Statistical significance was tested using a Mann-Whitney test. (D) Box and Whiskers plot showing proportions of proliferating CFSElow (left panel) and viable (right panel) cells within CXCR5^−^ PD-1^+^ non-Tfh cells present in culture after incubation with mDCs from Nt2 (orange, n = 4) and Nt1 (yellow, n = 5) Nts, NNs (green, n = 9), and healthy donors (blue, n = 5). Statistical significance was tested using a Mann-Whitney test. Error bars represent minimum to maximum values. (E) Proportions of IL-6 (left) and IL-12p70/p40 (right) producing cells in CD14^+^ Mo (top panels) and CD14^−^ CD11c^Hi^ HLA-DR^+^ mDCs (bottom panels) isolated from the blood of Nt2 (orange, n = 10), Nt1 (yellow, n = 6) Nts, and NN (green, n = 6) controllers cultured for 24 h in the presence of TLR8/7 or TLR2 agonists. The error bars represent minimum to maximum values. Values were corrected for basal levels present in media only (see Figure S5). The statistical significance between each condition among different patient cohorts was calculated using a Mann-Whitney test. ^∗^p < 0.05. (F) Box and Whiskers plot showing proportions of CXCR5^+^ PD-1^+^ (left plot) and CXCR3^Hi^ PD-1^+^ (right plot) CD4^+^ T cells from healthy donors induced in the presence of mDCs from Nt2 controllers in the presence of DMSO (n = 7) or STAT4 (n = 7) inhibitors. ^∗^p < 0.05, 2-tailed Wilcoxon matched-pairs signed rank test. Error bars represent minimum to maximum values. (G) Box plot and Whiskers plot showing fold change in the induction of Tfh-like cells defined as CXCR5^+^ PD-1^+^ (left panel) or Bcl-6^+^ PD-1^+^ cells (right panel) in the presence of mDCs isolated from healthy donors preincubated for 24 h in the presence of cytokines (IL-12 or IL-6 or TGF-β, n = 6 each) or TLR ligands (PGNSA or poly I:C, n = 6, or lipopolysaccharide [LPS], n = 5). Data were normalized to culture conditions with unstimulated mDCs. The error bars represent minimum to maximum values. ^∗^p < 0.05, two-tailed Wilcoxon matched-pairs signed rank test.

To assess the mDCs-CD4^+^ T cell interactions between our study cohorts in more detail, we analyzed whether there was a relation between frequencies of CXCR5^+^ PD-1^+^ peripheral blood Tfh (pTfh) in CD4^+^ T cells and the activation state of mDCs in Nt controllers. The levels of circulating pTfh were significantly correlated with the expression of the co-stimulatory molecule CD86 on peripheral blood mDCs in Nt controllers, suggesting reciprocal functional interactions between these two cell types (Figure 3A). To test this possibility, we used a functional co-culture assay previously developed by our group that tracks and quantifies DC-induced differentiation of naive CD4^+^ T cells into CXCR5^+^ PD-1^+^ Tfh-like cells (Figure S5C) (Martin-Gayo et al., 2017). Using this approach, we observed that *ex vivo* isolated mDCs from Nt2 were more effective in inducing Tfh-like cells compared to cells from Nt1 and NN patients and healthy donors (Figure 3B). These differences did not seem to be the consequence of altered proliferation or the viability of Tfh-like or non-Tfh-like CD4 T cells in these functional assays (Figures 3C and 3D).

Corresponding to the increased ability of mDC from Nt2 patients to prime Tfh-like cells *in vitro*, we also noted the increased secretion of interleukin-12 (IL-12), a cytokine with a critical role in Tfh priming (Yu et al., 2015, Schmitt et al., 2013), and IL-6 in mDCs from Nt2 at baseline and after stimulation with TLR8 and TLR2 ligands; enhanced expression of IL-12 and IL-6 was observed using protein and mRNA expression analysis techniques (Figures 3E, S8D, and S8E; Table S3). Such altered cytokine secretion activities were also noted in Mos from Nt2 and were obvious in comparison to Nt1 controllers, NN controllers, and healthy individuals. Notably, the secretion of transforming growth factor β (TGF-β), a cytokine previously associated with Tfh priming (Kobayashi et al., 2016), was not different in mDCs and Mos from the different study cohorts (Figure S8F).

Given that signal transducer and activator of transcription 4 (STAT4) is induced and activated by IL-12 and IL-6, we evaluated its possible role in mDC-mediated priming of Tfh-like cells. Neutralization of STAT4 by a small molecule inhibitor (Yang et al., 2003, Coon et al., 1999) significantly reduced co-stimulatory molecule expression in mDCs (Figure S5D) and translated into less efficient priming of CD4^+^ T cells toward a Tfh-like phenotype (Figures 3F and S5) without notably affecting cell viability (Figure S5B). In contrast, the inhibition of alternative putative upstream regulators for the transcriptional signatures of mDCs from Nt2 that were inferred with lower statistical significance levels in Nt2 patients did not have a significant effect on the function or maturation of mDCs (Figure S9). Notably, preconditioning of mDCs with IL-12 induced co-stimulatory molecule upregulation and enhanced Tfh priming (Figures 3G, S5A, and S5F). Similar results were observed when mDCs were incubated with IL-6 and TGF-β, while TLR ligands did not affect Tfh priming in this experimental system (Figures 3G and S5F). These data suggest that mDCs and Mos from Nt2 patients possess increased abilities to secrete IL-12 and prime mDCs to mediate Tfh cell differentiation.

### Tfh Cell and Th1 Gene Expression Signatures in CD4^+^ T from Nt2 Controllers

**Figure 4:**
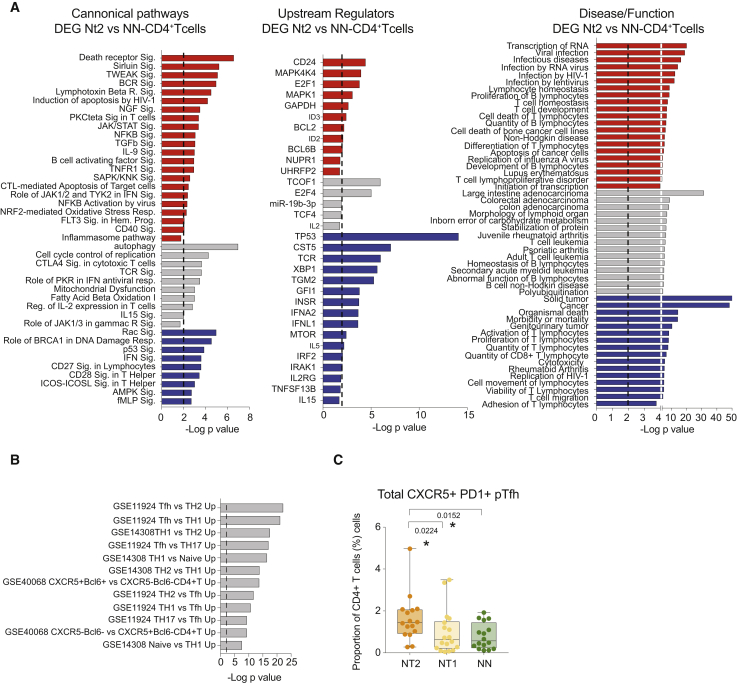
Circulating CD4^+^ T Cells from Nt2 Controllers Are Phenotypically and Transcriptionally Enriched in Tfh Cells (A) Selected significant canonical pathways (left), upstream regulators (center), and diseases and functions (right) predicted by IPA from DEGs (FDR p < 10e−5) in CD4^+^ T cells from Nt2 relative to Nt1 or NN controllers. Hits predicted as upregulated or downregulated by IPA are labeled in red (*Z* score > 0) and blue (*Z* score < 0), respectively; hits without inferred directional change are highlighted in gray. Statistical significance cutoff at −log p value = 2 has been highlighted with a discontinuous line. (B) Gene set enrichment analysis of DEGs from CD4^+^ T cells from Nt2 controllers compared to NNs using a pool of n = 18 public sets for human CD4 T cells. Statistical significance cutoff at −log p value = 2 has been highlighted with a discontinuous line. (C) Box and whiskers plot showing the proportions of circulating CXCR5^+^ PD-1^+^ CD4^+^ T cells within live CD4^+^ T cells in Nt2 (orange, n = 16), Nt1 (yellow, n = 18), and NN (green, n = 16) controllers. The error bars represent minimum to maximum values. Statistical significance was calculated by Mann-Whitney test and Bonferroni correction.

The close correlations between transcriptional signatures of mDCs and CD4^+^ T cells from Nt2 patients suggested that mDC-CD4^+^ T cell interactions may drive the evolution of neutralizing antibody breadth in Nt2 patients. A functional pathway analysis of the 1,191 DEGs (FDR p < 0.05) between CD4^+^ T cells from Nt2 and NN individuals indicated enrichment for transcripts involved in a wide spectrum of biological activities and mechanisms, including cellular activation, proliferation, and viral stress, but it failed to identify a unifying functional pattern distinguishing CD4^+^ T cells from Nt2 (Figures 4A, S6A, and S6B). The same was true for transcripts in CD4 T cells from Nt2 that were statistically associated with neutralizing antibody breadth. However, a comparison with publicly available gene sets suggested that CD4^+^ T cells from Nt2 patients were strongly enriched for genes involved in Tfh cells and Th1 responses (Figure 4B). This observation corresponded to increased proportions of circulating CXCR5^+^ PD-1^+^ pTfh cells in Nt2 compared to Nt1 and NN (Figure 4C). Consistent with our previous studies (Martin-Gayo et al., 2017), CXCR5^+^ PD-1^Lo^ precursors of both Th1 pTfh and non-TH1 pTfh seemed to contribute more significantly to the overall increase of pTfh in Nt2 controllers, particularly in the case of the Th1 pTfh subset (Figures S6C and S6D).

### Distinct Immune Signatures of Mos in Nt2 Controllers

**Figure 5:**
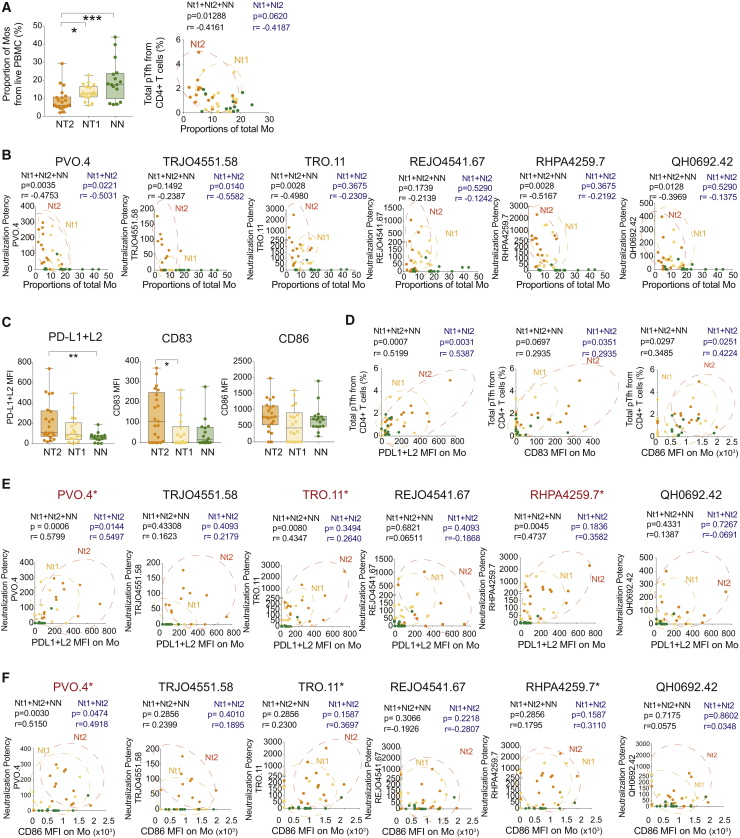
Distinct Numerical, Phenotypical, and Transcriptional Characteristics of Mos from Nt2 Controller (A) Left panel: box and whiskers plot showing the proportions of total CD14^+^ Mos among live PBMCs in Nt2 (orange, n = 21), Nt1 (yellow, n = 18), and NN (green, n = 16) controllers. The error bars represent minimum to maximum values. The lower panel shows the Spearman correlation between frequencies of total Mos and frequencies of total pTfh cells within CD4^+^ T cells in Nt controllers (Nt1, yellow; Nt2, orange). p and Spearman R values considering only Nt populations (blue) or all controllers including NNs (black) are shown on top of the plot. (B) Spearman correlation between proportions of total Mos and potency of antibody neutralization of the indicated n = 6 tier 2 and tier 3 pseudoviruses in Nt2 (orange) and Nt1 (yellow) and NN (green) controllers. p and Spearman R values considering only Nt populations (blue) or all patients including NNs (black) are shown on top of the plot. (C) Box and whiskers plots showing MFI of PD-L1+L2 (left), CD83 (center), and CD86 (right) in total Mos from Nt2 (orange, n = 21), Nt1 (yellow, n = 18), and NN controllers (green, n = 16). The error bars represent minimum to maximum values. ^∗∗^p < 0.01 (Mann-Whitney test). (D) Spearman correlations between the MFI of PDL1+L2 (left panels) CD86 (center panels), and CD83 (right panels) and plasma HIV-1 VL. p and Spearman R values considering only Nt populations (blue) or all patients including NNs (black) are shown on top of the plot. (E and F) Spearman correlations between the MFI of PD-L1 (E) and CD86 (F) with the potency of neutralization of 6 HIV-1 pseudoviruses virus. FDR-corrected p and Spearman R coefficient for only Nts (blue) or all patients (black) are shown.

**Figure 6:**
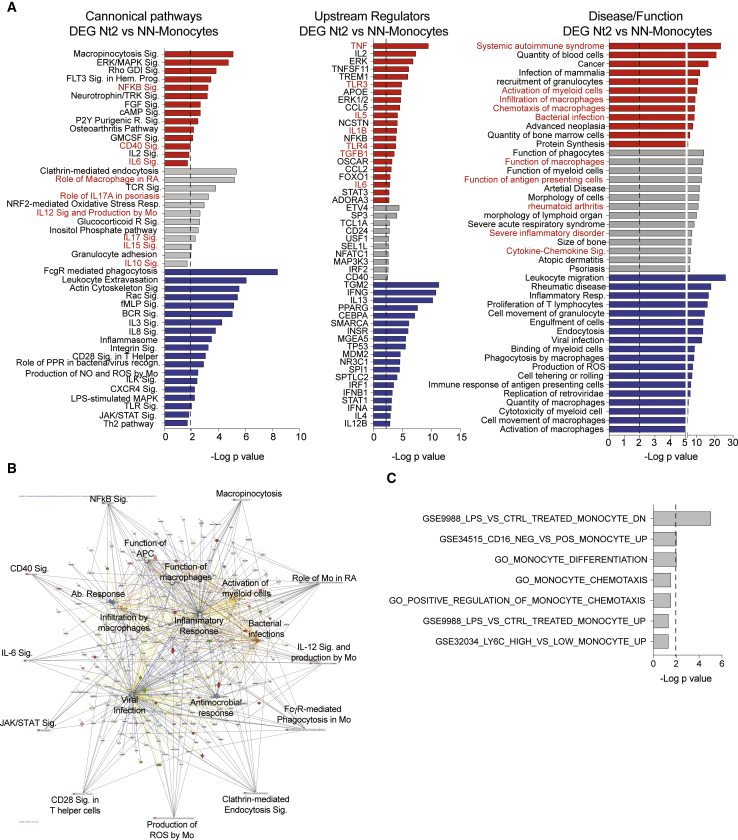
Inflammatory Transcriptional Signatures in Mos from Nt2 Controllers (A) Selected significant canonical pathways (left), upstream regulators (center), and diseases and functions (right) predicted by IPA from significant (FDR p < 0.05) DEGs in Mos from Nt2 versus NN controllers. Significance cutoff at −log p value = 2 was highlighted with a discontinuous line. Functions predicted to be upregulated, downregulated, or without predicted directional change are highlighted in red, blue, or gray, respectively. (B) Network analysis of predicted canonical pathways (outer edges) and disease and functions (inside) for DEG in Mos from NT2 patients. (C) Gene set enrichment analysis of significant DEGs from Nt2 versus NN controllers in n = 19 public gene sets related to Mo function and migration. Data correspond to −log FDR-corrected p values from selected predicted pathways.

In our subsequent analysis, we focused on the possible role of Mos in contributing to the generation of antibodies with increased neutralizing breadth in HIV controllers. We observed that among all controllers, Mo populations were associated with the relative fractions of circulating pTfh, and with a higher potency of neutralization for 3 of 6 HIV-1 pseudoviruses. Notably, proportions of total (CD14^+^), classical (CD14^Hi^ CD16^−^), transitional (CD14^Hi^ CD16^Dim^), and nonclassical (CD14^Lo^ CD16^Hi^) Mos tended to be decreased in the blood from Nt2 controllers, but appeared to display a more mature phenotype, defined by the elevated expression of CD83 and PD-L1 and PD-L2 (Figures 5C and S8B). PD-L1 and PD-L2 expression levels on Mos correlated with the potency of neutralization of 3 of 6 HIV-1 pseudoviruses preferentially recognized by plasma antibodies from Nt2 (Figure 5E), while CD86, a co-stimulatory molecule that was not differentially expressed on Mos from Nt2 patients, was only weakly related to neutralization potency (Figure 5F). Expression intensities of CD83, CD86, PD-L1, and PD=-L2 were also positively associated with the proportion of circulating Tfh cells within the entire controller study cohort (Figure 5D). There was no significant association between co-stimulatory molecule expression on the membrane of Mos and plasma HIV-1 VL (Figure S5C), suggesting that the specific surface phenotype of Mos in Nt2 is not driven by viremia. In addition to these phenotypic differences, a computational analysis of DEGs that distinguished Nt2 Mos from NN patients revealed immune signatures involved in activation, chemotaxis, inflammatory cytokine signaling, and TLR-dependent immune recognition (Figures 6A and 6B). These pathways play important roles in regulating inflammatory responses, antiviral immune responses and infiltration, and trafficking of Mos and macrophages (Figures 6B and 6C). Notably, a dedicated linear regression analysis between Mo gene expression signatures and corresponding neutralizing antibody breadth identified transcripts involved in Ag presentation, immune regulation, and cytokine-dependent cell interaction as the top correlative variables (Figures S7A and S7B). These results indicated that distinct phenotypic and transcriptional signatures of Mos are components of the Nt2-defining immune network.

## Discussion

Typically, bnAbs develop in individuals with high-level viremia and increased immune activation; however, HIV-1-specific antibodies with high neutralizing breadth have also been detected in rare subgroups of HIV-1 controllers with low or undetectable VLs in the absence of ART. Here, we have identified distinct transcriptional and functional features of primary mDCs, Mos, and CD4^+^ T cells in a subgroup of HIV-1 controllers (Nt2), who developed higher neutralizing Ab breadth against HIV-1. In this specific patient population, we noted a transcriptional program in mDCs characterized by a robust upregulation of inflammatory genes and activation of pathways supporting Tfh polarization, which was coupled with a higher functional ability of mDCs to prime Th1-biased PD-1^+^ Tfh-like cells *in vitro*. This functional and phenotypical profile of mDCs was associated with increased numbers of circulating Tfh-like cells, with a distinct immune signature of Mos, which were reduced in numbers but displayed a more inflammatory gene expression pattern. In addition, multiple layers of connections between individual modules of DEGs from these different cell populations were also observed in this specific patient population. These studies identify what appear to be integrative, multi-compartment immune networks that are selectively detectable in a subgroup of HIV controllers with enhanced neutralizing antibody breadth and are likely to be functionally involved in enabling and facilitating the evolution of such humoral immune responses in the absence of ongoing high-level viral replication. However, it is important to mention that this immunological profile was not a necessary prerequisite for the development of neutralizing antibodies in HIV controllers, since we observed none of the described immune features in an alternative group of controllers, called Nt1, exhibiting an equal breadth of neutralizing antibody responses, although antibodies from Nt2 controllers seemed to have a higher potency in neutralizing some HIV-1 tier 2 pseudoviruses. In addition, differences in the potency of neutralization may reflect differential epitope targeting by antibodies produced in Nt2 versus Nt1 Nts and deserves further study. These results suggest that mechanisms supporting the evolution of neutralizing antibody breadth in controllers are complex, may vary among different patients, and are likely to involve interrelated immune networks between different immune cell types.

Our studies suggest that in Nt2 Nt controllers, the development of neutralizing antibody responses may be facilitated by mDCs possessing improved Tfh-priming function, which was associated with (and likely causally related to) numerically increased Tfh/Th1 CD4^+^ T cells in these patients. A Tfh/Th1 polarization of circulating CD4^+^ helper cells has been linked to functionally enhanced humoral immune responses in a variety of disease contexts, both during natural disease conditions and in vaccine recipients (Baiyegunhi et al., 2018), and functional studies suggest the ability of these cells to support Ig class switching to IgG and to secrete cytokines enabling proper B cell maturation (Craft, 2012, Crotty, 2011). Although we were unable to analyze CD4^+^ Th cells from tissues in our study patients, our findings are compatible with and suggest more active germinal center responses against HIV-1 in the Nt2 patients, since proportions of circulating pTfh can be considered a biomarker of active germinal center reaction (Heit et al., 2017, Simpson et al., 2010). An important aspect of future studies will be the identification of the epitope specificity of HIV-1 antibodies in Nt1 and Nt2 patients; previous investigations suggest preferential antibody targeting of the viral envelope V3-glycan region (González et al., 2018, Freund et al., 2017) in HIV-1 controllers. The identification of the viral env epitopes most frequently targeted by bnAbs in HIV-1 controllers may indeed be highly informative in this context.

One important unresolved question in this study is the absence of distinguishable immune features in Nt1 patients, which also mount antibodies with higher neutralizing breadth relative to the control cohorts of NN controllers. This supports prior observations of a considerable immunological heterogeneity between individual controllers, despite their seemingly identical clinical phenotype. Based on previous investigations, HIV-1 controllers with extremely strong, intermediate, or absent HIV-1-specific T cell responses have been described and associated with distinct transcriptional patterns in CD4^+^ (Vigneault et al., 2011) and CD8^+^ T cells (Chowdhury et al., 2018). In this regard, we found that the frequencies of HLA-B alleles linked to protection against disease progression (Gao et al., 2005) and associated with improved cytotoxic CD8^+^ T cell function (Kiepiela et al., 2004) were significantly higher in Nt1 than in Nt2 controllers; these differences in the expressed HLA class I isotype may exert important immunoregulatory effects through interactions with major histocompatibility complex (MHC) class I receptors on Mos and DCs (Bashirova et al., 2014). Moreover, all of the Nt controllers from our study were characterized by significantly higher VLs than NN controllers (Figure S1), but plasma VLs tended to be highest in the Nt2 patients among all of the controllers. These observations may suggest that a slightly increased viral Ag exposure leads to higher levels of immune activation in Mos, B cells, CD4 T cells, and mDCs from Nt2 patients, and may facilitate the evolution of HIV-1-specific antibody breadth during low viremia conditions; higher VLs have been repeatedly associated with higher neutralizing Ab breadth in HIV-1 progressors (Locci et al., 2013, Crotty, 2014). Nevertheless, it remains remarkable that the Nt1 subset of HIV-1 controllers is seemingly able to mount high levels of neutralizing antibody breadth in the absence of distinct immune signatures of immune activation or elevated circulating Tfh responses; this suggests that viral mechanisms, such as higher sequence diversity, may have a predominant influence on the breadth of neutralizing antibodies. Clearly, a better understanding of the specific mechanisms enabling elevated neutralizing breadth in this patient population represents an important aspect of future investigations.

To our knowledge, our investigation represents one of the few studies in which the role of circulating Mos was specifically assessed in the context of HIV-1-specific antiviral immune responses. Unexpectedly, we noted quantitative reductions in circulating Mo counts, a finding that may be attributable to Mo tissue segregation, possibly into lymph nodes in which Mos may interfere and support the germinal center reaction. We observed transcriptional signatures of Mos that were suggestive of enhanced migratory and chemotactic activities, in addition to a more activated immune profile, suggesting a distinct immune trafficking behavior of these cells in Nt2 patients. Coupled with the elevated expression of co-stimulatory markers, it is possible that the specific immune signature in Nt2 Mo cells may play a previously underappreciated role in the evolution of neutralizing antibodies, and clearly warrants future investigation. Our work represents a study identifying integrated, multi-compartment signatures in HIV controllers with increased neutralizing antibody breadth, and provides information about cellular mechanisms involved in effective and broad humoral responses that may be informative in the design of future neutralizing antibody-based preventive vaccines.

## STAR★Methods

### Key Resources Table

**Table:** 

REAGENT or RESOURCE	SOURCE	IDENTIFIER
**Antibodies**		

Alexa Fluor 700 anti-human CD14 Antibody	BioLegend	Cat 325614; RRID: AB_830687
Brilliant Violet 570 anti-human CD16 Antibody	BioLegend	302035; RRID: AB_10915988
APC anti-human CD40 Antibody	BioLegend	334310; RRID: AB_2260153
Brilliant Violet 605 anti-human CD86 Antibody	BioLegend	374214; RRID: AB_2734430
PE anti-human CD83 Antibody	BioLegend	305308; RRID: AB_314516
PE/Cy7 anti-human HLA-DR Antibody	BioLegend	307616; RRID: AB_493588
Pacific Blue anti-human CD11c Antibody	BioLegend	301626; RRID: AB_10662381
PE/Dazzle 594 anti-human CD274 (PD-L1) Antibody	BioLegend	329732; RRID: AB_2616889
PE/Dazzle 594 anti-human CD273 (PD-L2) Antibody	BioLegend	329618; RRID: AB_2716089
Brilliant Violet 570 anti-human CD4 Antibody	BioLegend	317445; RRID: AB_2561582
Brilliant Violet 605 anti-human CD3 Antibody	BioLegend	317322; RRID: AB_2561911
PerCP/Cyanine5.5 anti-human CXCR5 Antibody	BioLegend	356910; RRID: AB_2561819
PE anti-human CD183 (CXCR3) Antibody	BioLegend	353706; RRID: AB_10962912
APC anti-human CD279 (PD-1) Antibody	BioLegend	329908; RRID: AB_940475
APC/Cyanine7 anti-human CD19 Antibody	BioLegend	302218; RRID: AB_314248
Brilliant Violet 421 anti-human Bcl-6	BD Biosciences	563363; RRID: AB_2738159
Biotin anti-human CD275 (ICOS-L) Antibody	BioLegend	309406; RRID: AB_528729
LIVE/DEAD Fixable Blue Dead Cell Stain Kit	Life technologies	LS23105

**Bacterial and Virus Strains**		

HIV-1 pseudovirus AC10.0.29 Env plasmid/DNA	NIH AIDS Reagent P	11024
HIV-1 pseudovirus RHPA4259.7 Env plasmid/DNA	NIH AIDS Reagent P	11036
HIV-1 pseudovirus THRO4156.18 Env plasmid/DNA	NIH AIDS Reagent P	11037
HIV-1 pseudovirus REJO4541.67 Env plasmid/DNA	NIH AIDS Reagent P	11035
HIV-1 pseudovirus WITO4160.33 Env plasmid/DNA	NIH AIDS Reagent P	11033
HIV-1 pseudovirus TRO11 Env plasmid/DNA	NIH AIDS Reagent P	11023
HIV-1 pseudovirus SC422661.8 Env plasmid/DNA	NIH AIDS Reagent P	11058
HIV-1 pseudovirus QH0692.42 Env plasmid/DNA	NIH AIDS Reagent P	11018
HIV-1 pseudovirus CAAN5342.A2 Env plasmid/DNA	NIH AIDS Reagent P	11410
HIV-1 pseudovirus PVO.4 Env plasmid/DNA	NIH AIDS Reagent P	11022
HIV-1 pseudovirus TRJO4551.58 plasmid/DNA	NIH AIDS Reagent P	11034

**Biological Samples**		

PBMC from HIV-1 controller cohorts	Ragon Institute repository CIDB	n/a

**Chemicals, Peptides, and Recombinant Proteins**		

Recombinant Human IL-12 p70	Peprotech	200-12
Recombinant Human IL-6	Peprotech	200-06
Recombinant Human TGF-β1	Peprotech	100-21C
Lisofylline	Enzo	BML-LP102-0010
AS1842856	Calbiochem	344355
PGN-SA TLR2 Agonist	InvivoGen	tlrl-pgns2
Poly I:C TLR3 agonist	Sigma-Aldrich	P1530-25MG
TLR7/8 Agonist (CL097)	InvivoGen	tlrl-c97

**Critical Commercial Assays**		

mirVanaTM Isolation Kit	Life Technologies	AM1560
Agencourt RNAClean XP SPRI beads	Beckman Coulter	A63987
SMARTer® Ultra Low Input RNA Kit for Illumina® Sequencing-HV, 96 reactions	Clontech	634828
Nextera XT DNA Sample Preparation Kit, 96	Illumina	FC-131-1096
CD1c (BDCA-1) + Dendritic Cell Isolation Kit, human	Miltenyi Biotec	130-119-475
CD141 (BDCA-3) MicroBead Kit, human	Miltenyi Biotec	130-090-512
Naive CD4+ T Cell Isolation Kit II, human	Miltenyi Biotec	130-094-131
Naive B Cell Isolation Kit II, human	Miltenyi Biotec	130-091-150

**Deposited Data**		

RNA-seq	Gene Expression Omnibus	GSE141498

**Experimental Models: Cell Lines**		

TZM.bl cell line	NIH AIDS Reagent P	8129

**Software and Algorithms**		

Ingenuity Pathway Analysis	QIAGEN Bioinformatics	IPA
DEseq2 package	Bioconductor	DEseq2
WGCNA package	R	WGCNA
Dynamic tree cut algorithm	Bioinformatics	Langfelder et al. (2008)
Circlize package	CRAN	circlize

### Lead Contact and Materials Availability

Dr. Xu G. Yu, M. D. is the Lead contact of the study. Any inquiry related to reagents or resources related to the present study and can be contacted via email to the following addresses: xyu@mgh.harvard.edu.

This study did not generate new unique reagents.

#### Experimental model and subject details

HIV-1 controllers who had maintained < 2000 copies/ml HIV-1 VL for a median of 5 years in the absence of antiretroviral therapy, with (neutralizers, Nt, n = 46, median VL 302.5 copies/ml, range 24,800-20 copies/ml; median CD4 counts 729.5 cells/ml, range 1,603-385 cells/ml, median age 51 years, range 28-68, 84% male) or without (non-neutralizers, NN, n = 15; median VL 48 copies/ml, range 4180-20 copies**/**ml; median CD4 counts 825 cells/ml range 1684-407 cells/ml, median age 49, range 39-66, 86% male) broad neutralizing Ab against HIV-1 in plasma were recruited for this study. Samples from patients who met all of the above criteria except for a single viral blip were also included in this study.

All subjects gave written informed consent and the study was approved by the Institutional Review Board of Massachusetts General Hospital/Partners Healthcare.

### Method Details

#### Analysis of the neutralizing breadth of HIV-1-specific antibodies

HIV-1 neutralization breadth was defined in a luciferase-Tzm-bl cellbased pseudovirus neutralization assay previously described (Sarzotti-Kelsoe et al., 2014) against a panel of Env-pseudoviruses derived from 9 Clade B and C Tier 2 and two Tier 3 neutralization sensitivities: AC10.0.29^∗^, RHPA4259.7^∗^, THRO4156.18^∗^, REJO4541.67^∗^, WITO4160.33^∗^, TRO.11^∗^, SC422661.8^∗^, QH0692.42^∗^, CAAN5342.A2^∗^ and Tier 3: PVO.4^∗^ and TRJO4551.58^∗^. The Clade B isolates are denoted by the superscripts ^∗^. The assay quantifies a reduction in the expression of the reporter gene luciferase in TZM.bl cells after one single-round of infection. Plasma samples were inactivated at 56°C for 1h and 3-fold serial dilutions of these samples were incubated by duplicate with HIV-1 Env- Pseudoviruses during 1h at 37°C. Subsequently, TZM.bl cells were added in the presence of medium containing 11 μg/ml DEAE-dextran and incubated at 37°C for 48 h. Expression of the Luciferase reporter was determined with Bright-Glo luciferase reagent (Promega). The serum dilution at which a 50% of reduction of relative light units (RLU) was observed (relative to background-corrected RLU numbers in control cell wells, 50% inhibitory dose [ID50]) was used to define the neutralization titer. Neutralization potency was defined as plasma dilutions in which at least 50% inhibition of infection after background subtraction at a 1:20 dilution was observed. The neutralization breadth was defined as the percentage of the 11 isolates neutralized by each plasma sample. All samples were screened for non-HIV-1-specific neutralization using murine leukemia viruspseudotyped virions.

#### Flow cytometry phenotypical analysis and cell sorting

Live human Lin^-^ CD14^-^ CD11c^+^ HLADR^+^ mDCs, CD14^+^ monocytes, CD4^+^ CD3^+^ T cells and CD19^+^ B cells from n = 45 HIV controller neutralizers and n = 15 non-neutralizers were sorted by flow cytometry using a FACS Aria II sorter (BD Biosciences) for RNaseq analysis. Pre-gating on viability dye was used to select viable cells. For phenotyping studies, *ex vivo* and cultured PBMC were stained with LIVE/DEAD cell blue viability dye (Invitrogen, Carlsbad, CA) and different panels of monoclonal antibodies. (CD14, CD16, CD40, CD86, CD83, HLA-DR, CD11c, ICOS-L and PDL1+L2(Biolegend). mDCs were identified from bulk PBMCs as a population of viable CD14^-^ lymphocytes expressing high levels of CD11c and HLA-DR. For phenotypical characterization of CD4+ T cells, anti-human CD4 (BD), CD3, CXCR5, CXCR3, PD-1 (Biolegend) was used. Finally, for identification of cultured B cells, we used an anti-CD19 mAb (Biolegend). Samples were analyzed on a Fortessa cytometer (BD Biosciences, San Jose, CA). Data were analyzed with FlowJo software (Tree Star).

#### Gene expression analysis by RNA-Seq

Total RNA was obtained from Lin^-^ CD14^-^ CD11c^+^ HLADR^+^ mDCs, CD14^+^ monocytes, CD4^+^ CD3^+^ T cells and CD19^+^ B sorted from peripheral blood of neutralizer (n = 45) and non-neutralizer (n = 15) controllers using the *mir*Vana™ Isolation Kit (Life Technologies™). Subsequently, RNA-Seq libraries from each sorted Tfh-like population were generated as previously described (Trombetta et al., 2014). Briefly, whole transcriptome amplification (WTA) and tagmentation-based library preparation was performed using SMART-seq2 (Trombetta et al., 2014), followed by sequencing on a NextSeq 500 Instrument (Illumina). Sequences were then aligned using the Hg38 human genome database by Bowtie 2 (Langmead and Salzberg, 2012), and transcripts per million (TPM) values were obtained for each sample by RNA-Seq using Expectation-Maximization (RSEM) (Li and Dewey, 2011). TPM values were then normalized among all samples using the upper quantile normalization method.

#### Magnetic cell isolation for functional assays

Total CD19^-^ BDCA1^+^ and BDCA3^+^ conventional dendritic cells (mDCs) were purified from total PBMC by immunomagnetic enrichment as previously described (Martin-Gayo et al., 2015) (purity >90%). Naive CD4^+^ T cells and naive CD27^-^ B cells were isolated using immunomagnetic negative selection kits (Miltenyi Biotec, Naive CD4^+^ T cell Isolation Kit II and Naive B cell Isolation Kit II human), leading to a cell of purity >95%. MS and LD columns and/or the AutoMACS (Miltenyi Biotec) system were used for cell isolation.

#### *In vitro* DC-based priming of Tfh-like cells

Freshly isolated naive CD4^+^ T cells were co-cultured with autologous naive B cells in the presence or absence of allogeneic mDCs from different patient cohorts in 96 round-bottom well plates for 6-7 days as previously described (Martin-Gayo et al., 2017). Presence of CXCR5^+^ PD-1^+^ Bcl6^+^ Tfh-like cells was analyzed by flow cytometry at the end of the culture. In some experiments, PBMCs were pre-cultured for 24h in the presence of either 0.2ng/ml cytokines (IL-12 or IL-6 or TGFβ; Peprotech) or 2 μg/ml TLR ligands (PGNSA, Poly I:C, LPS) or small molecule inhibitors for STAT-4 (0.6 μM Lisofylline; Enzo) or Y FOX I (33nM ASI842856; Calbiochem) inhibitors prior to the isolation of mDCs. When appropriate, matching concentration of DMSO was used to supplement control culture conditions for each inhibitor.

#### Cytokine secretion assays

PMBCs from neutralizer and non-neutralizer controllers or healthy individuals were cultured either in media or in the presence of TLR2 or TLR8 agonists. After 24h, vesicle transport inhibitors brefeldin A (BioLegend) and Golgi Stop (BD Biosciences) were added the culture media following the manufacturer’s instructions and cells were cultured for 5 additional h. Subsequently, cells were washed with 1x PBS and intracellular expression of IL-12 and IL-6 in CD14^-^ CD11c^Hi^ HLADR^+^ mDCs and CD14^+^ Monocytes was evaluated by flow cytometry.

### Quantification and Statistical Analysis

#### Computational data analysis of RNA-Seq data

Unsupervised hierarchical clustering analysis of normalized TPM was conducted to discriminate clusters of patients. Differential expressed gene analyses were performed with the DEseq2 package on the pairwise comparisons of every two groups of interest (Love et al., 2014). False-discovery rates (FDR) with varying thresholds were used for the control of multiple comparison issues. Gene co-expression networks were constructed and analyzed using WGCNA package (Langfelder and Horvath, 2008). Power values for soft thresholds were determined automatically by the package. The robustness of the procedure was ensured by using bi-weight mid-correlation to quantify the correlation between genes and by using a signed hybrid network using the TOM (topological overlapping matrix) method for adjacency metrics, as recommended by the software author (https://horvath.genetics.ucla.edu/html/CoexpressionNetwork/Rpackages/WGCNA/). Gene modules were identified by the dynamic tree cut algorithm, and the total number of modules were pre-determined to be 2 to avoid co-expressing genes getting clustered into similar minor modules (Langfelder and Horvath, 2008). The correlation coefficients between genes from different cells types were calculated directly for different groups of patients, [using the cor() function from the WGCNA package].p values were corrected by FDR for multiple comparisons. In some cases, Spearman correlation analysis was performed for transcripts of each cell type to identify genes whose expressions are highly correlated with neutralizing antibody breadth. Finally, circos plots reflecting connections between gene modules and individual genes among different cell types were created using the Circlize package (Gu et al., 2014).

#### Statistics

Significance of phenotypic differences between the cells from different patient cohorts were assessed using Mann Whitney U tests or Wilcoxon matched-pairs signed-rank test. When possible a, statistical analysis was corrected for multiple comparisons using a Kruskal-Wallis test with post hoc Dunn’s test, the Bonferroni correction or False Discovery Rate (FDR).

### Data and Code Availability

The RNA-seq data used for the current study has been deposited and is available at the Gene Expression Omnibus (GEO accession GSE141498).

## Supplemental Information

Document S1. Figures S1–S9 and Tables S1–S4

Document S2. Article plus Supplemental Information
