# Supplementary material for: Immunological Fingerprints of Controllers Developing Neutralizing HIV-1 Antibodies
Source: Cell Rep. 2020 Jan 28;30(4):984–996.e4. doi: 10.1016/j.celrep.2019.12.087 (PMC6990401; doi:10.1016/j.celrep.2019.12.087)
Supplement: Document S1. Figures S1–S9 and Tables S1–S4 [file mmc1.pdf]

Cell Reports, Volume *30*

Supplemental Information

Immunological Fingerprints of Controllers Developing Neutralizing HIV-1 Antibodies

Enrique Martin-Gayo, Ce Gao, Hsiao Rong Chen, Zhengyu Ouyang, Dhohyung Kim, Kellie E. Kolb, Alex K. Shalek, Bruce D. Walker, Mathias Lichterfeld, and Xu G. Yu

Supplemental Figure 1

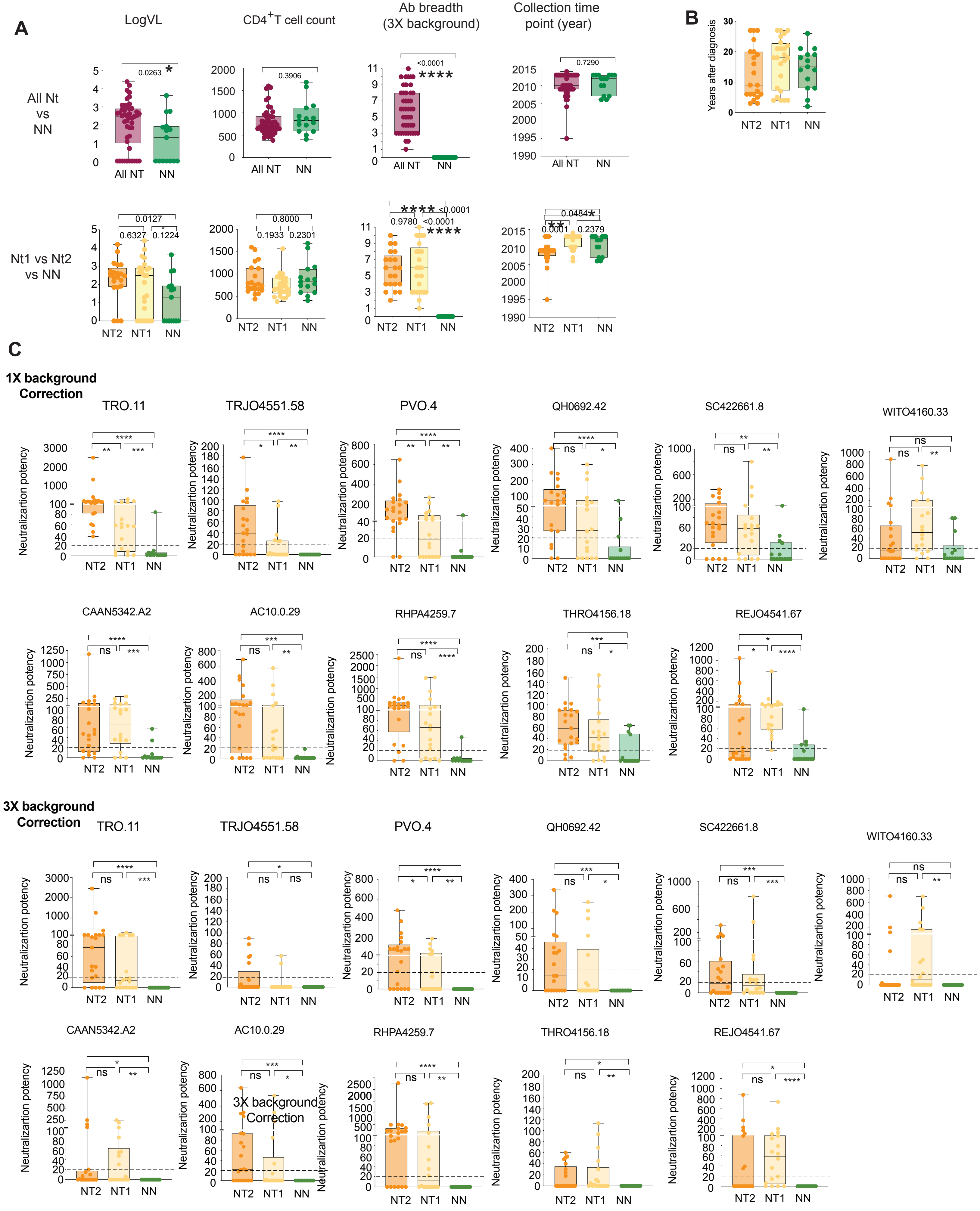

Supplemental Figure 1. Clinical characteristics of Nt2 controllers (Related to Figure 1). (A): Box and Whiskers plots showing plasma HIV-1 viral load (VL), $CD4^{+}$ T cell counts (CD4), neutralizing antibody breadth (Ab. breadth) and sample collection time point for all Neutralizers (left, n=46) or Nt1 (n=25) versus Nt2 (n=21) neutralizer controllers (right) compared to non-neutralizers (n=15). Error bars represent Min to Max values. Statistical significance was calculated using a chi square test (*p<0.05; **p<0.01; ****p<0.0001). (B) Box and Whiskers plots showing time after diagnosis (years) of Nt2 (n=21), Nt1 (n=25) and non-neutralizer (n=15) controllers. Error bars represent Min to Max values. (C): Box and Whiskers plots showing potency of antibody neutralization from plasma from Nt2 (orange, n=21), Nt1 (yellow, n=18) and non-neutralizer (green, n=13) against the indicated eleven HIV-1 tier 2 and tier 3 pseudoviruses after 1X (upper plots) and 3X (lower plots) subtraction of background levels. Error bars represent Min to Max values. Statistical significance was calculated using a two tailed Mann Whitney test (*p<0.05; **p<0.01). Potency of neutralization was considered positive for those values superior to 1/20 dilution indicated by a discontinuous line .

Supplemental Figure 2

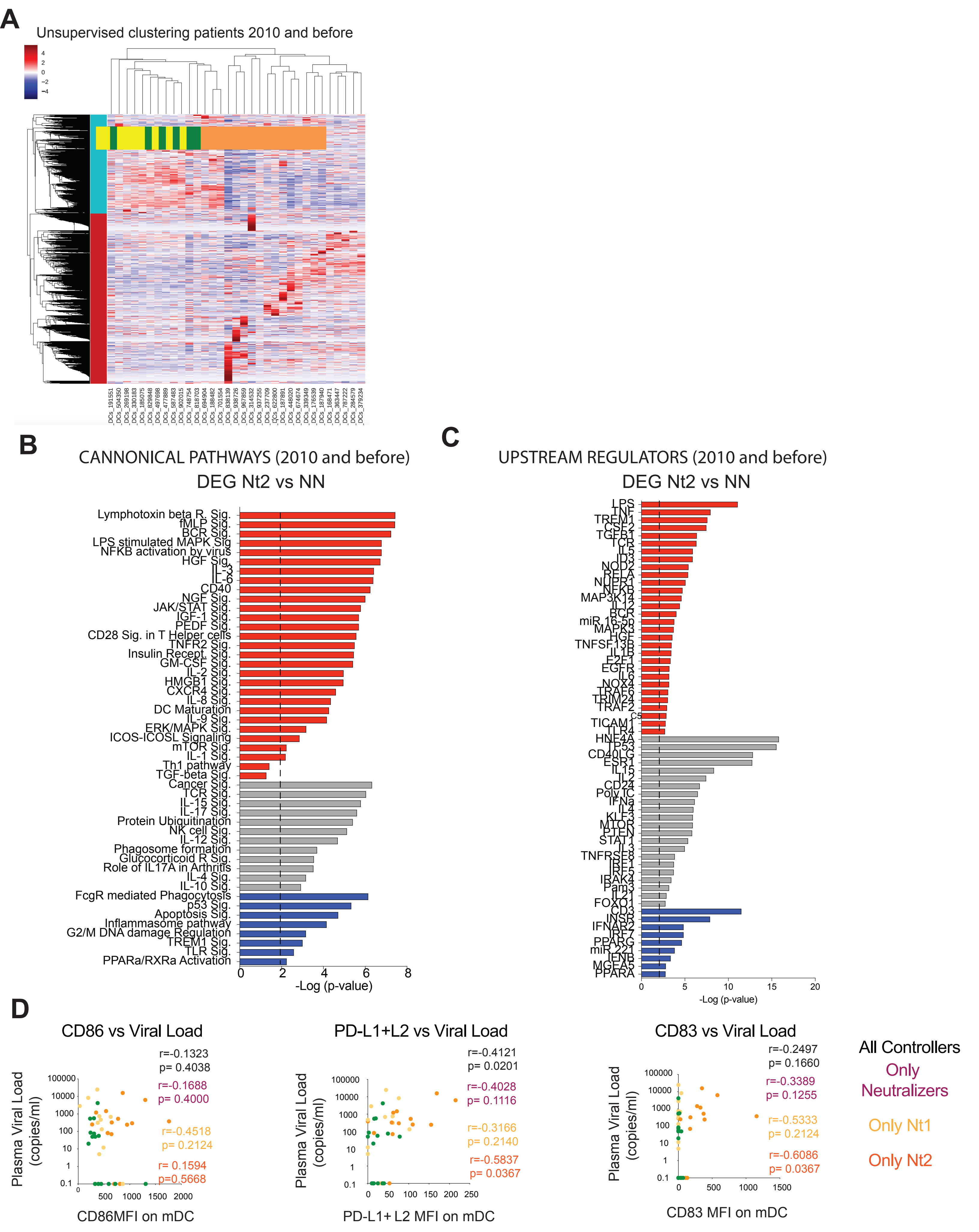

Supplemental Figure 2. Transcriptional characteristics of Nt2 controllers and correlations to viral load (Related to Figure 1). (A): Unsupervised clustering based on the expression of 13,239 in mDCs from neutralizer and non-neutralizer (green) controller blood samples collected in 2010 and earlier time points defining Nt2 (orange) and Nt1 (yellow) subgroups. (B-C): Predicted canonical pathways (B) and upstream regulators (C) of differentially expressed genes (FDR p<10e-5) between mDCs from Nt2 controllers versus non-neutralizers using samples from 2010 and earlier time points. Pathways that are significantly upregulated, downregulated or with undetermined directional changes are highlighted in red, blue and grey, respectively. (D): Correlation of HIV-1 Plasma viral load with mean fluorescence intensity (MFI) of CD86 (left), PDL1+L2 (middle) and CD83 (right) considering Nt1 (yellow), Nt2 (Orange) and NN (green) or all (black) controllers. Spearman FDR-corrected P and R values considering different patient groups are highlighted on the corresponding color on each plot.

Supplemental Figure 3

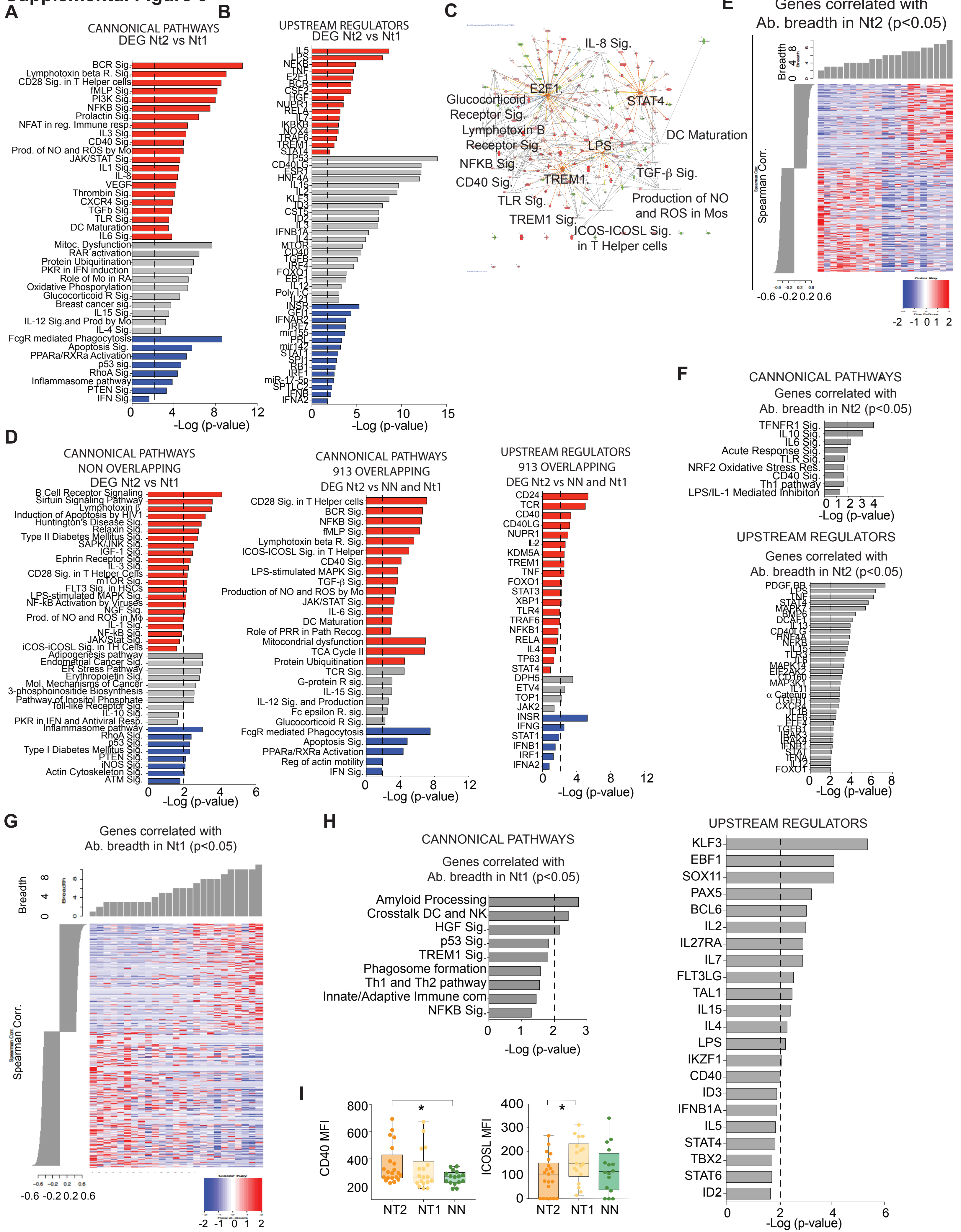

Supplemental Figure 3. Differential transcriptional signatures of mDCs from Nt2 compared to Nt1 neutralizer controllers (Related to Figure 1). (A-B): Selected Cannonical (correct spelling in figure) pathways (A) and upstream regulators (B) predicted by Ingenuity Pathway Analysis for differentially expressed genes (FDR p<10e-5) between mDCs from Nt2 compared to Nt1 neutralizer controllers. Predictions of upregulation, downregulation or with undetermined directional change are represented in red, blue and grey, respectively. Significance cut off at -log p value=2 was highlighted with a discontinuous line. (C): Network analysis of selected upstream regulators and canonical pathways from DEG between mDCs from Nt2 compared to Nt1 neutralizer controllers. (D): Canonical pathways of DEG between mDCs from Nt2 vs Nt1 controllers which do not overlap with DEG of mDCs from Nt2 compared to NN controllers. Analysis of canonical pathways and upstream regulators for overlapping 913 DEG between mDC from Nt2 vs Nt1 and NN are shown on the right. Significance cut off at -log p value=2 was highlighted with a discontinuous line. (E-G): Heatmap reflecting expression levels of genes significantly (nominal p<0.05) correlated with neutralizing antibody breadth in mDCs from the blood of Nt2 (E) or Nt1 (G) controllers. (F-H): List of selected canonical pathways and upstream regulators predicted by Ingenuity Pathway Analysis for genes correlated with neutralizing antibody breadth in mDCs from Nt2 (F) and Nt1 (H) controllers. Grey represents pathways without a predicted z score. (I): Box and Whiskers plots showing Mean of Fluorescence Intensity (MFI) of CD40 (left) and ICOSL (right) in mDCs from Nt2 (orange, n=21) and Nt1 (yellow, n=18) neutralizer and non-neutralizer (green, n=16) controllers. Error bars represent Min to Max values. Statistical significance was calculated using a Mann Whitney test (*p<0.05, **p<0.01).

Supplemental Figure 4

Corr.CD86 MFI mDC vs Neutralization Potency

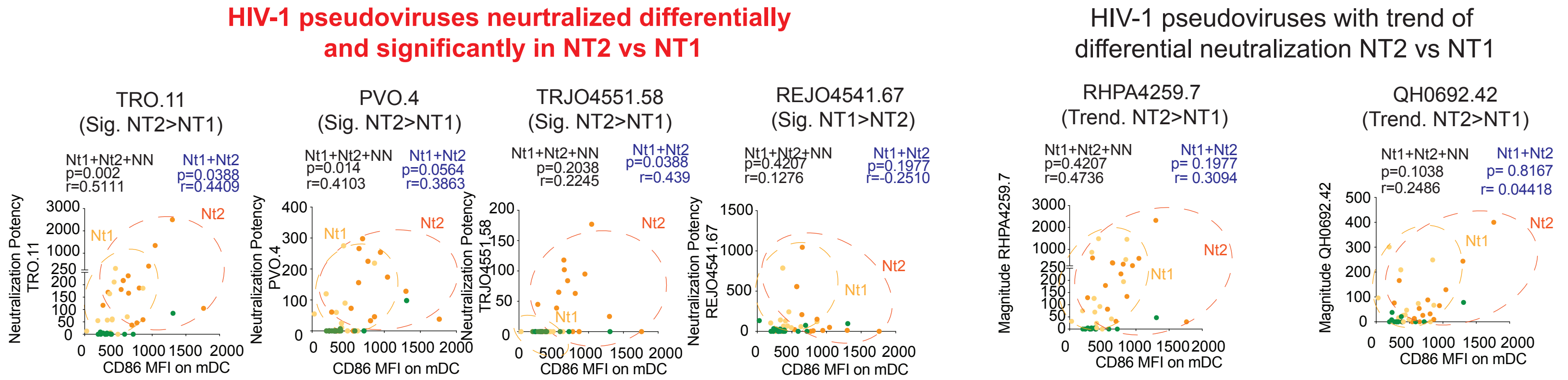

Corr.CD83 MFI mDC vs Neutralization Potency

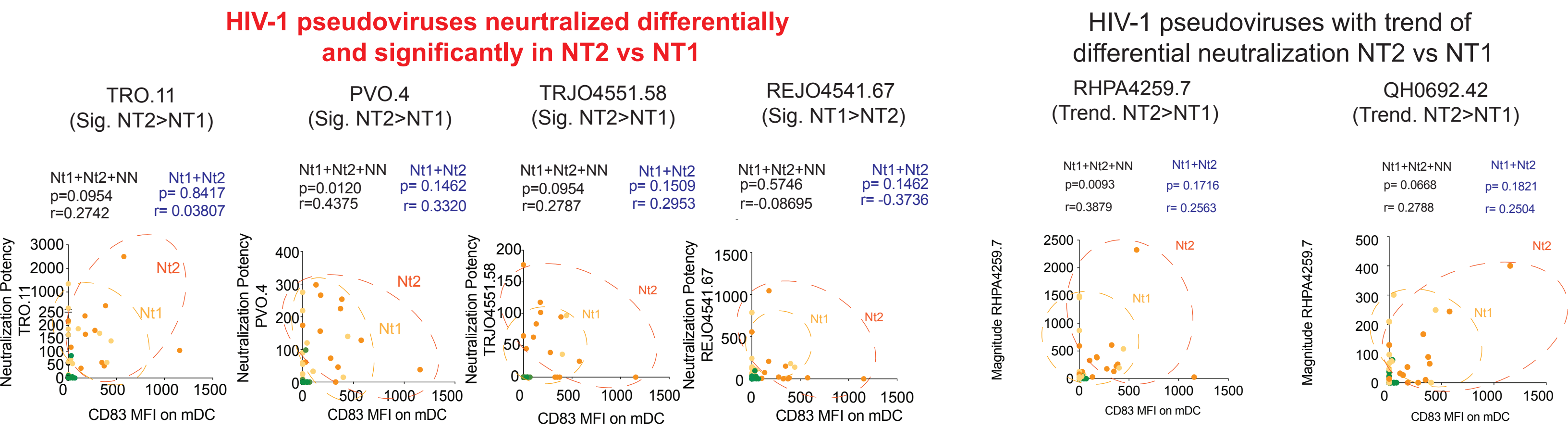

Corr. PDL1+L2 MFI mDC vs Neutralization Potency

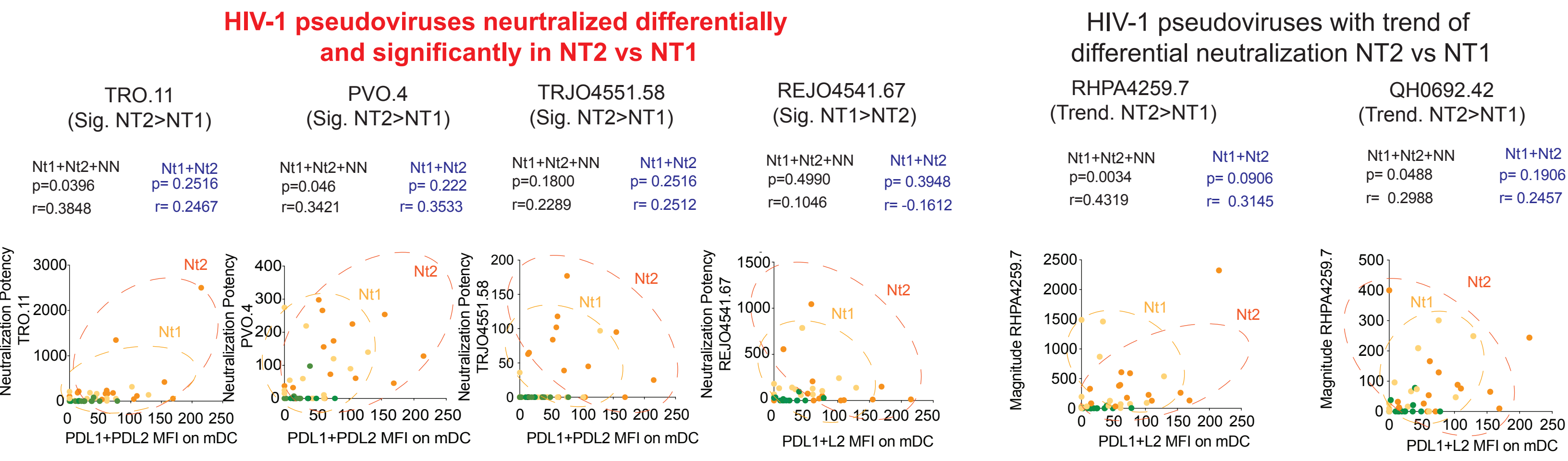

Supplemental Figure 4. Associations between Antibody Neutralization potencies and DC phenotype (Related to Figure 1). Correlation of CD86, CD83 and PDL1+L2 MFI with potency of neutralization of 6 out of 11 HIV-1 Tier 2 and Tier3 HIV-1 viruses with different neutralization values (significant highlighted in red, trend in black) between plasma from Nt2 and Nt1 patients (also see Supplemental Table 1). FDR-corrected P and spearman R values considering only Nt1 (yellow) and Nt2 (orange) neutralizer populations or all patients including non neutralizers (green) are highlighted in blue and black, respectively.

Supplemental Figure 5

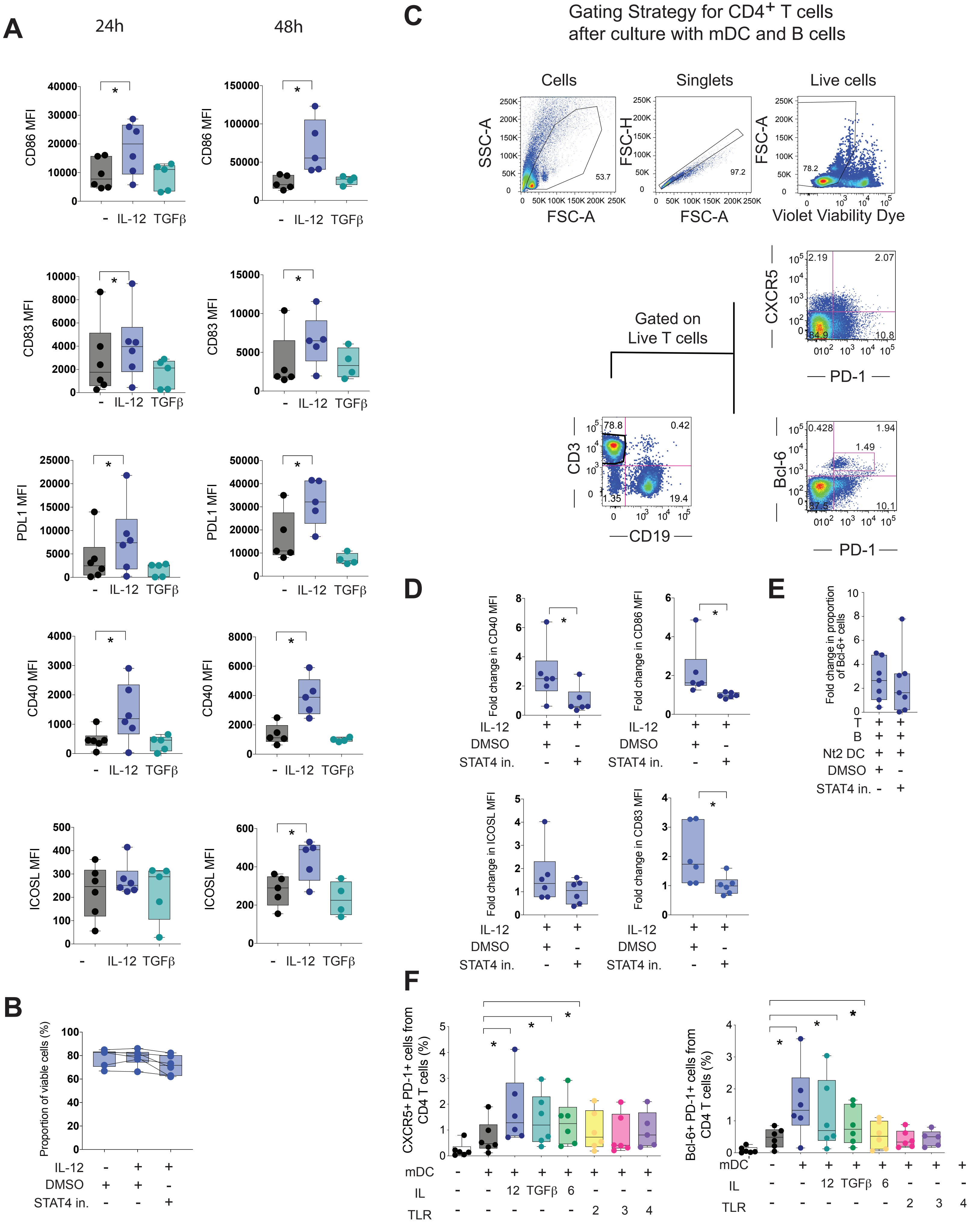

Supplemental Figure 5. Impact of IL-12 stimulation in mDC maturation and Tfh priming function (Related to Figure 3). (A): Box and Whiskers plots showing Mean Fluorescence Intensity of CD86, CD83 and PD-L1 on mDCs cultured for 24 (left panels) and 48h (right panels) in the absence (grey, n=6 24h, n=5 48h) or the presence of IL-12 (blue, n=6 24h, n=5 48h) or TGFbeta (green, n=5 24h, n=4 48h). Error bars represent Min to Max values. Statistical significance was calculated using a two-tailed Wilcoxon matched pairs test (*p<0.05). (B): Box and Whiskers plots showing proportions of viable cells (defined as life-dead viability dye negative cells) included in $CD14^-$ $CD11c^{Hi}$ $HLADR^+$ mDCs in PBMCs cultured in the absence or the presence of IL-12 and DMSO or STAT4 small molecule inhibitors. Error bars represent Min to Max values. (C): Flow cytometry gating strategy used to identify $CXCR5^+$ $PD\text{-}1^+$ and Bcl-6+ DP-1+ T cells after co-culture with allogeneic mDCs from different patient cohorts. The data correspond to a representative experiment. flow cytometry dotplots shows selection of single live lymphocytes and further gating on $CD3^+$ $CD19^-$ cells and analysis of co-expression of CXCR5 and PD-1 (upper panel) or Bcl-6 and PD-1(lower panel) defining Tfh like $CD4^+$ T cells induced *in vitro* in the presence of autologous B cells and allogeneic mDCs cultured as described in Figure 3. (D): Box and Whiskers plots showing fold change in Mean Fluorescence Intensity (MFI) of CD40, CD86, ICOSL and CD83 on mDCs cultured for 24h in the presence of IL-12 alone (n=6) or in combination with a STAT4 inhibitor (n=6). Error bars represent Min to Max values. Statistical significance was calculated using a two-tailed Wilcoxon matched pairs test (*p<0.05). (E): Box and Whiskers plots showing proportions of $Bcl\text{-}6^+$ $PD1^+$ $CD4^+$ T cells induced from naïve $CD4^+$ T cells from healthy donors in the presence of autologous B cells and allogeneic mDCs from Nt2 controllers precultured for 24h in the presence of either DMSO (n=7) or a STAT4 inhibitor (n=7). Error bars represent Min to Max values. (F): Box and Whiskers plots showing raw proportions of $CXCR5^+$ (left plot) or $Bcl\text{-}6^+$ (right plot) $PD\text{-}1^+$ Tfh-like cells from $CD4^+$ T cells present in culture after 5 days of incubation with autologous naïve B cells alone (white, n=6) or with allogeneic mDCs previously treated for 24h in the presence of media (grey n=6), IL-12 (blue, n=6), TGF-beta (dark green, n=6), IL-6 (light green, n=6), TLR2L (yellow, n=6), TLR3L (pink, n=6) and TLR4L (violet, n=5). Error bars represent Min to Max values. Statistical significance against cultured treated with unstimulated DC was calculated using a wilcoxon test (*p<0.05).

Supplemental Figure 6

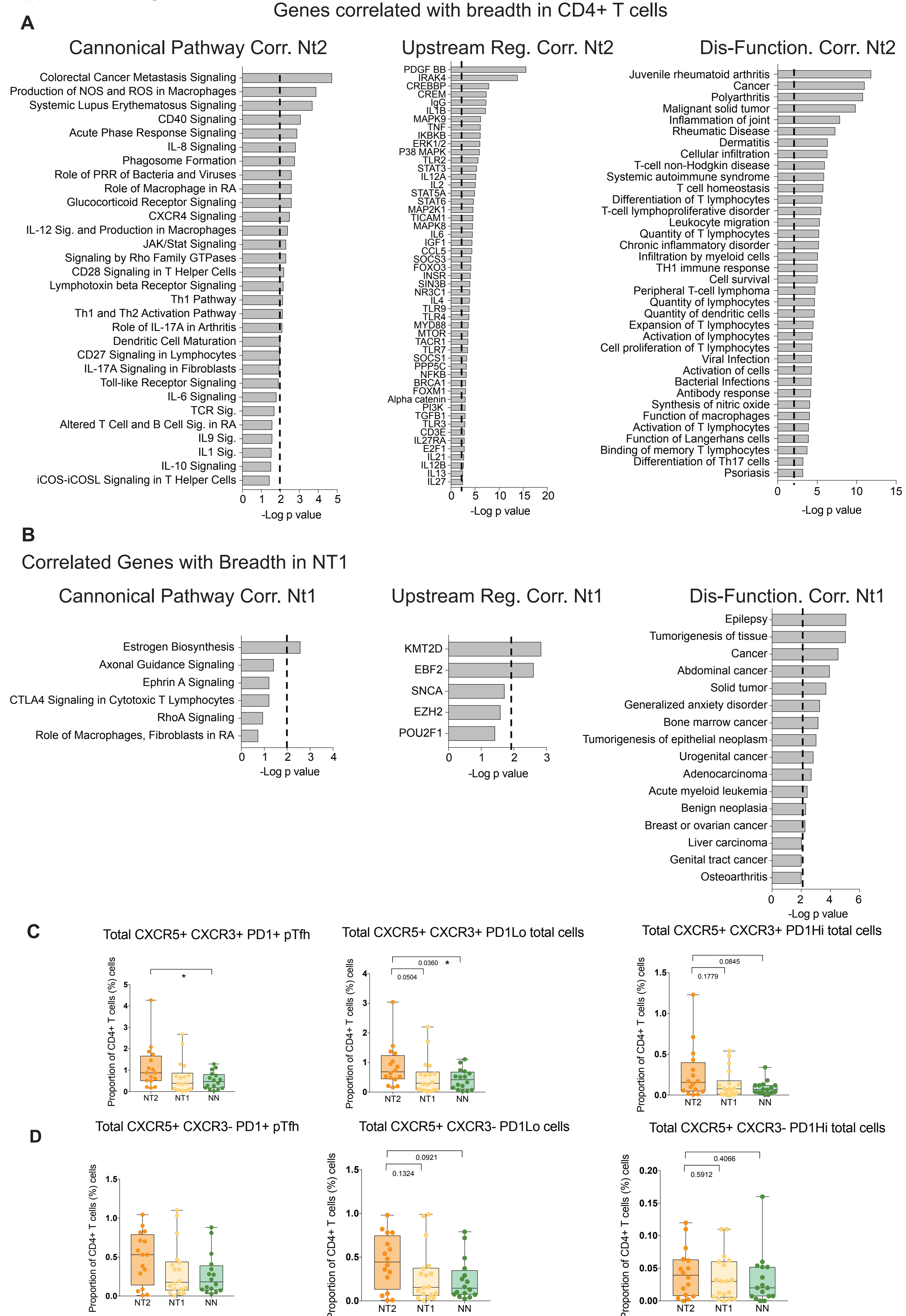

Supplemental Figure 6 (related to figure 4). Transcriptional and phenotypical features of $CD4^+$ T cells from neutralizer controller subgroups. (A-B): Selected canonical pathways (left), upstream regulators (middle) and diseases and function (right) from genes significantly (nominal $p<0.05$) correlated with neutralizing antibody breadth in circulating $CD4^+$ T cells from Nt2 (A) and Nt1 (B) controllers. Significance cut off at -log p value=2 was highlighted with a discontinuous line. (C-D): Box and Whiskers plots showing proportions of Th1 $CXCR3^+$ (C) and Non Th1 $CXCR3^-$ (D) total $CXCR5^+$ $PD\text{-}1^+$ (left), $CXCR5^+$ $PD\text{-}1^{Lo}$ (middle) and $CXCR5^+$ $PD\text{-}1^{Hi}$ (right) pTfh among live PBMC from the blood of Nt2 (orange, n=16) and Nt1 (yellow, n=18) neutralizer and non-neutralizer (green, n=16) controllers. Error bars represent Min to Max values. Statistical significance was calculated with a two-tailed Mann Whitney test.

Supplemental Figure 7

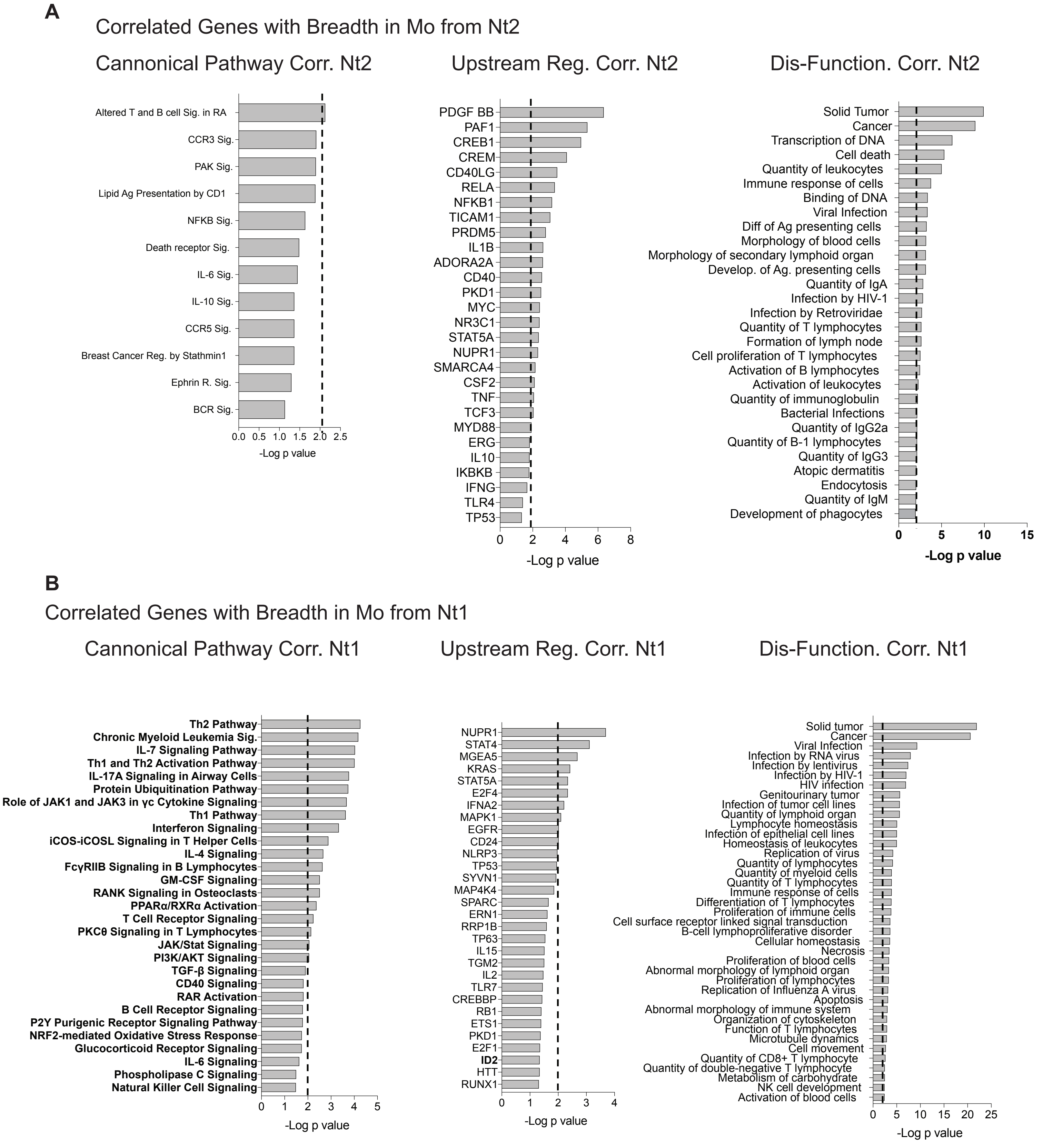

Supplemental Figure 7 (related to Figure 5). Transcriptional characterization of monocytes from Nt2 neutralizer controllers. (A-B): Selected canonical pathways (left), upstream regulators (middle) and diseases and function (right) from genes significantly (nominal $p<0.05$) correlated with neutralizing antibody breadth in circulating monocytes from Nt2 (A) and Nt1 (B) controllers. Significance cut off at -log p value=2 was highlighted with a discontinuous line.

Supplemental Figure 8

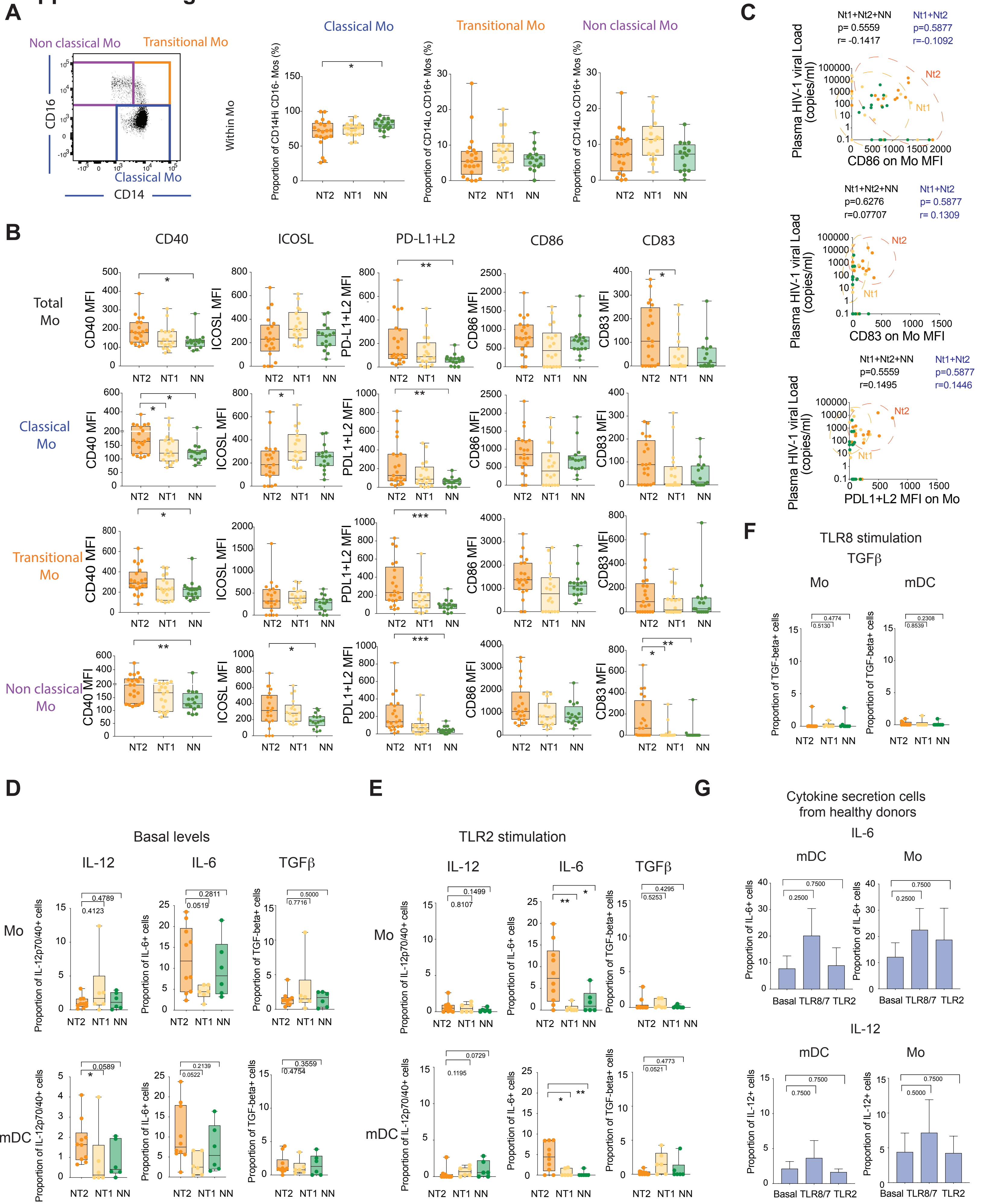

Supplemental Figure 8 (related to Figure 5). Phenotypical characterization and cytokine production of monocytes from Nt2 neutralizer controllers.
(A): Left panel: Representative flow cytometry analysis of levels of CD16 versus CD14 on gated total $CD14^{+}$ monocytes defining classical $CD14^{Hi}$ $CD16^{-}$, transitional $CD14^{+}$ $CD16^{+}$ and non-classical $CD14^{Lo}$ $CD16^{Hi}$ Mo subsets are shown on the left. Right panel: Box and Whiskers plots showing proportions of each Mo subset from total $CD14^{+}$ Mo in Nt2 (orange, n=21) and Nt1 (yellow, n=18) neutralizer and non-neutralizer (green, n=16) controllers. Error bars represent Min to Max values. Statistical significance was calculated using a two-tailed Mann Whitney test. (B): Box and Whiskers plots showing Mean of Fluorescence Intensity of surface CD40, ICOSL, PDL1+L2, CD86 and CD83 in total or in indicated $CD14^{+}$Mo subsets from Nt2 (orange, n=21) and Nt1 (yellow, n=18) neutralizer and non-neutralizer (green, n=16) controllers. Error bars represent Min to Max values. Statistical significance was calculated using a two-tailed Mann Whitney test. (C): Spearman correlations of plasma HIV-1 viral loads and mean of fluorescence intensity (MFI) of CD86, CD83 and PDL1+L2 on Mo from Nt1 (yellow) and Nt2 (orange) neutralizers and NN (green) controllers. Spearman R and FDR-corrected P values considering all patient groups (black) or only neutralizers (blue) are shown. (D-E-F) Box and Whiskers plots showing proportions of IL-6, IL-12 and TGF-beta producing cells in $CD14^{+}$ Mo and $CD14^{-}$ $CD11c^{Hi}$ $HLADR^{+}$ mDCs isolated from the blood of Nt2 (orange, n=10) and Nt1 (yellow, n=6) neutralizers and non-neutralizer (green, n=6) controllers cultured for 24h in the presence of media (D, Basal levels) or in the presence of TLR2 (E, data were corrected for background basal levels) or TLR8 agonists (F, only TGF-beta, also corrected for basal levels). Error bars represent Min to Max values. Statistical significance was calculated using a Mann Whitney test (G): Proportions of cells secreting IL-6 (upper plots) and IL-12 (lower plots) in mDCs (left) and Mos (right) from n=3 healthy donors after 24h of culture in the presence of media (basal) or TLR8/7 or TLR2 agonists. Data correspond to Mean and SEM values.

Supplemental Figure 9

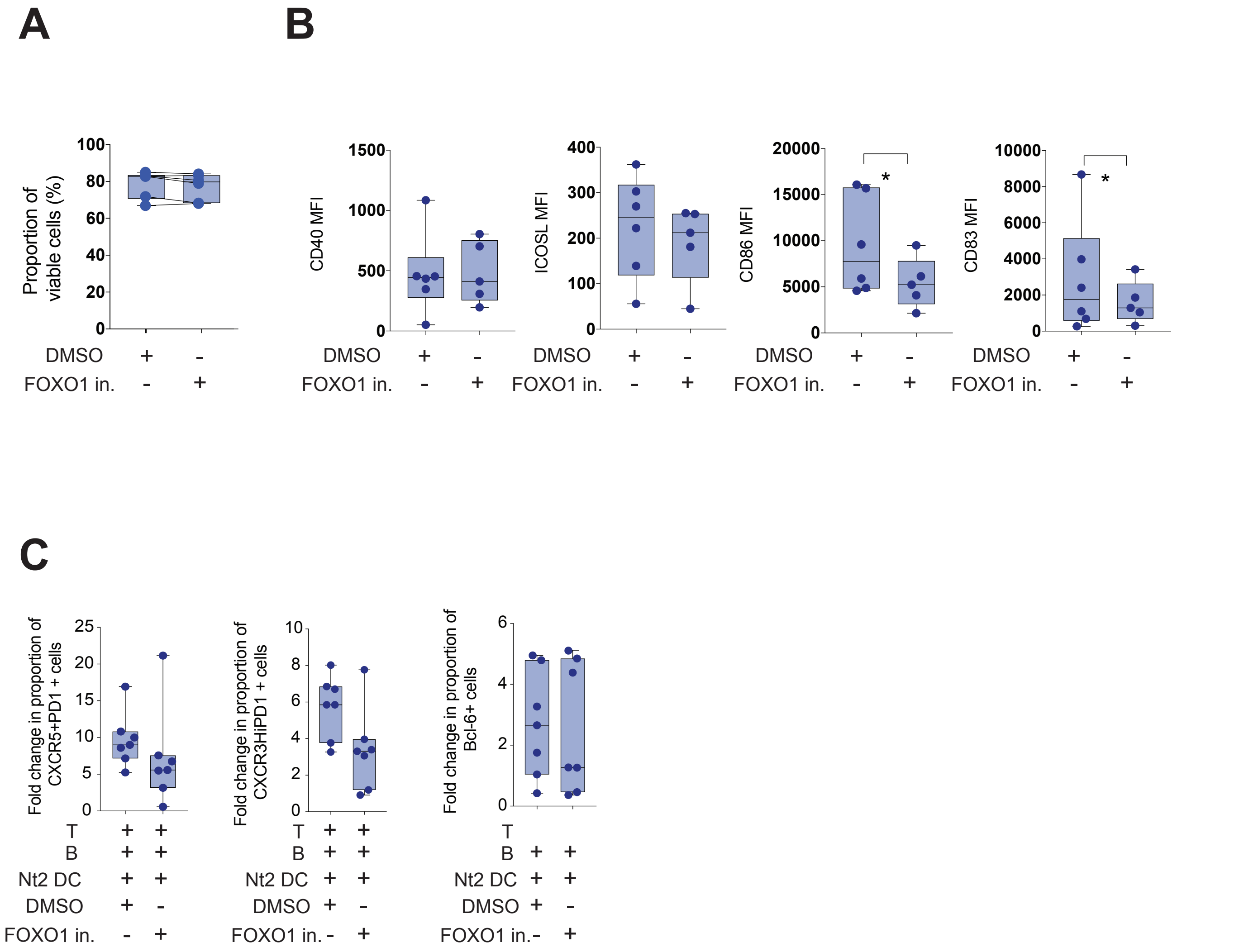

Supplemental Figure 9 (related to Figure 3). Impact of FOXO 1 activity on mDCs maturation and Tfh priming function. (A): Box and Whiskers plots showing proportions of viable cells (defined as life-dead viability dye negative cells) included in $CD14^-$ $CD11c^{Hi}$ $HLADR^+$ mDCs in PBMCs cultured in the absence or the presence of DMSO (n=6) or a FOXO 1 small molecule inhibitor (n=6). Error bars represent Min to Max values. (B): Box and Whiskers plots showing Mean of Fluorescence Intensity of CD40, ICOSL, CD86 and CD83 in mDCs cultured in the presence of either DMSO (n=6) or a FOXO I (n=5) inhibitor. Error bars represent Min to Max values. Statistical significance was calculated using a two-tailed Wilcoxon matched pairs test (*p<0.05). (C): Box and Whiskers plots showing proportions of $CXCR5^+$ $PD\text{-}1^+$ (left panel), $CXCR3^{Hi}$ $PD\text{-}1^+$ (middle panel) and $Bcl6^+$ $PD1^+$ (right panel) $CD4^+$ T cells induced from naïve $CD4^+$ T cells from healthy donors in the presence of autologous B cells and allogeneic mDCs from Nt2 controllers precultured for 24h in the presence of either DMSO (n=7) or a FOXO I inhibitor (n=7). Error bars represent Min to Max values.

HIV-1 pseud.	Median Neut Nt2 patients	Median Neut Nt1 patients	Median Neut NN patients	Nt2 vs NN P value Nom/Bonf	Nt2 vs Nt1 P value Nom/ Bonf	Nt1 vs NN P value Nom/ Bonf
QH0692.42	76	27	0	0.0001/ 0.0002	0.0785/0.157	0.011/0.022
SC422661.8	67	59	0	0.0018/0.0036	0.5082/1.0164	0.0047/0.0094
PVO.4	140	20	0	0.0001/ 0.0002	0.0066/ 0.0132	0.0064/0.0128
TRO.11	170	57.5	0	0.0001/ 0.0002	0.0014/0.0028	0.0005/0.001
AC10.0.29	108	22	0	0.0001/ 0.0002	0.1427/0.2854	0.0012/0.0024
RHPA4259.7	250	66	0	0.0001/ 0.0002	0.1237/0.2474	0.0001/0.0002
THRO4156.18	58	42	0	0.0002/0.0004	0.2911/0.5822	0.0101/0.0202
REJO4541.67	15	106	0	0.0419/0.0838	0.0351/0.0702	0.0001/0.0002
TRJO4551.58	39	0	0	0.0001/ 0.0002	0.0204/0.0408	0.0074/0.0148
WITO4160.33	15	51.5	0	0.2445/0.4890	0.0566/0.1132	0.002/0.004
CAAN5342.A2	47	66.5	0	0.0001/ 0.0002	0.9389/1.8778	0.0001/0.0002

Median Potency of neutralization of plasma tested against 11 HIV-1 pseudoviruses after 1X background subtraction. Nominal (Nom) or Bonferroni-corrected (Bonf) p values are shown.

HIV-1 pseud.	Median Neut Nt2 patients	Median Neut Nt1 patients	Median Neut NN patients	Nt2 vs NN P value Nom/Bonf	Nt2 vs Nt1 P value Nom/ Bonf	Nt1 vs NN P value Nom/ Bonf
QH0692.42	14	0	0	0.0005/ 0.001	0.2078/0.4156	0.0228/0.0456
SC422661.8	18	13.5	0	0.0008/ 0.0016	0.8645/1.729	0.0003/0.0006
PVO.4	86	0	0	0.0001/0.0002	0.0409/ 0,0818	0.0096/0.0192
TRO.11	77	13.5	0	0.0001/0.0002	0.0596/ 0.1192	0.0007/0.0014
AC10.0.29	22	0	0	0.0005/0.001	0.1769/ 0.3538	0.0228/0.0456
RHPA4259.7	139	12	0	0.0001/0.0002	0.1938/0.3876	0.0017/0.0034
THRO4156.18	0	1	0	0.0196/0.0392	0.6061/1.2122	0.0036/0.0072
REJO4541.67	0	59	0	0.0196/0.0392	0.1327/0.2654	0.0001/0.0002
TRJO4551.58	0	0	0	0.0286/0.0572	0.1/0.2	0.4968/0.9936
WITO4160.33	0	11.5	0	0.1317/0.2634	0.0641/0.1282	0.0017/0.0034
CAAN5342.A2	0	3	0	0.0113/0.0226	0.5797/1.1594	0.0226/0.0072

Median Potency of neutralization of plasma tested against 11 HIV-1 pseudoviruses after 3X background subtraction. Nominal (Nom) or Bonferroni-corrected (Bonf) p values are shown.

Supplemental Table 1 (related to Figure 1). Antibody Neutralization data from study patient cohorts.

	comparison	estimate	statistic	p.value	parameter	conf.low	conf.high	method	alternative	p.adj
1	B mod 1 v B mod 2	0.0476003147128245	2532.37378858122	1	1	0.0428310908759316	1	1-sample proportions test with continuity correction	greater	1
2	B mod 1 v CD4 mod 1	0.0521405049396268	36255.0594086734	1	1	0.0508121478497527	1	1-sample proportions test with continuity correction	greater	1
3	B mod 1 v CD4 mod 2	0.187321859837688	16199.3112557946	1	1	0.185208901001644	1	1-sample proportions test with continuity correction	greater	1
4	B mod 1 v DC mod 1	0.155102208694937	22950.2353976336	1	1	0.153197325134346	1	1-sample proportions test with continuity correction	greater	1
5	B mod 1 v DC mod 2	0.150079080995588	29562.8392442561	1	1	0.148387893264057	1	1-sample proportions test with continuity correction	greater	1
6	B mod 1 v Mono mod 1	0.0236267931048339	28375.9687527592	1	1	0.0225228149256374	1	1-sample proportions test with continuity correction	greater	1
7	B mod 1 v Mono mod 2	0.0748376908229514	9635.42579077175	1	1	0.071999726304842	1	1-sample proportions test with continuity correction	greater	1
8	B mod 2 v CD4 mod 1	0.11814383343366	17805.950352243	1	1	0.115919356623754	1	1-sample proportions test with continuity correction	greater	1
9	B mod 2 v CD4 mod 2	0.121723115881557	21342.3683103259	1	1	0.11969068217132	1	1-sample proportions test with continuity correction	greater	1
10	B mod 2 v DC mod 1	0.356375455583963	397.869875737586	1	1	0.353468913644174	1	1-sample proportions test with continuity correction	greater	1
11	B mod 2 v DC mod 2	0.0226356930529561	52061.9100896798	1	1	0.021832612330073	1	1-sample proportions test with continuity correction	greater	1
12	B mod 2 v Mono mod 1	0.271270659510018	2311.77587575174	1	1	0.267504145970763	1	1-sample proportions test with continuity correction	greater	1
13	B mod 2 v Mono mod 2	0.0167672313761894	10194.2156132605	1	1	0.0152051008678072	1	1-sample proportions test with continuity correction	greater	1
14	CD4 mod 1 v CD4 mod 2	0.12432446723611	307609.432556768	1	1	0.123787923042754	1	1-sample proportions test with continuity correction	greater	1
15	CD4 mod 1 v DC mod 1	0.51899771020486	72751.8200013243	0	1	0.518206554934803	1	1-sample proportions test with continuity correction	greater	0
16	CD4 mod 1 v DC mod 2	0.113639514017151	434703.17672917	1	1	0.113188047186649	1	1-sample proportions test with continuity correction	greater	1
17	CD4 mod 1 v Mono mod 1	0.32519616548247	10443.7936500297	1	1	0.324159939483628	1	1-sample proportions test with continuity correction	greater	1
18	CD4 mod 1 v Mono mod 2	0.0974934651620087	92324.9534214211	1	1	0.0965262711413087	1	1-sample proportions test with continuity correction	greater	1
19	CD4 mod 2 v DC mod 1	0.650954565079059	373103.243591722	0	1	0.650273898681085	1	1-sample proportions test with continuity correction	greater	0
20	CD4 mod 2 v DC mod 2	0.569666284064996	216713.786402854	0	1	0.569030360863551	1	1-sample proportions test with continuity correction	greater	0
21	CD4 mod 2 v Mono mod 1	0.145138416364537	174357.283127749	1	1	0.144436718737479	1	1-sample proportions test with continuity correction	greater	1
22	CD4 mod 2 v Mono mod 2	0.206297049782119	45223.7723068777	1	1	0.205105722988346	1	1-sample proportions test with continuity correction	greater	1
23	DC mod 1 v DC mod 2	0.193148737546691	289049.342196853	1	1	0.192656311720864	1	1-sample proportions test with continuity correction	greater	1
24	DC mod 1 v Mono mod 1	0.27448874985762	41910.3008721151	1	1	0.273623787586843	1	1-sample proportions test with continuity correction	greater	1
25	DC mod 1 v Mono mod 2	0.162883521176314	72787.8467206335	1	1	0.161826998905064	1	1-sample proportions test with continuity correction	greater	1
26	DC mod 2 v Mono mod 1	0.0608959184935705	409664.851917698	1	1	0.0604795686036169	1	1-sample proportions test with continuity correction	greater	1
27	DC mod 2 v Mono mod 2	0.227414246077541	46443.5354824437	1	1	0.226334474002863	1	1-sample proportions test with continuity correction	greater	1
28	Mono mod 1 v Mono mod 2	0.0260628400080594	94937.9636726719	1	1	0.0254295204902565	1	1-sample proportions test with continuity correction	greater	1

Supplemental Table 2 (related to Figure 2). List of p and correlation coefficient for each Gene module comparison across B cells, CD4 T cells, DCs and Mos.

Transcriptional levels of inflammatory cytokines in Dendritic cells.

Cytokine trainscripts in DEG from DC	Log2FC Nt2 vs NN	Nom p/FDR p Nt2 vs NN	Log2FC Nt2 vs Nt1	Nom p/FDR p Nt2 vs Nt1
CXCL10 (IP-10)	161.6	0.014/0.045	1.267	0.0301/0.072
IL6	240.9	9.85e10-9/0.00078	0.67	0.285/0.414
IL12A	226.15	6.31e10-9/0.00054	0.780	0.710/NA
IL12B	113.110	0.09633/0.19377	0.9047	0.3036/0.761
CCL5(Rantes)	19.966	9.85e10-9/0.000786	640.419	0.949/0.9785

Transcriptional levels of inflammatory cytokines in Monocytes

Cytokine trainscripts in DEG from Mo	Log2FC Nt2 vs NN	Nom p/FDR p Nt2 vs NN	Log2FC Nt2 vs Nt1	Nom p/FDR p Nt2 vs Nt1
CXCL10 (IP-10)	-0.6408	0.3155/0.9274	0.3150	0.7387/0.8877
IL6	2.0942	0.2577/0.9274	160.092	0.4207/NA
IL12A	1.2317	0.5025/0.9274	0.7800	0.7108/NA
IL12B	1.4622	0.6269/0.9275	0.9047	0.3036/0.7614
CCL5(Rantes)	0.8719	0.0998/0.7896	640.419	0.9495/0.97851

Transcriptional levels of Tfh-related cytokines in CD4+ T cells

Cytokine transcripts in DEG from CD4+T cells	Log2FC Nt2 vs NN	Nom p/FDR p Nt2 vs NN	Log2FC Nt2 vs Nt1	Nom p/FDR p Nt2 vs Nt1
CXCL13	0.1169	0.0369/0.970551	0.2319	0.9732/NA
IL-21	1.086	0.3879/NA	0.7508	0.7297/NA
CD40LG	-0.2721	0.1216/0.2614	395.452	0.043/0.113
IFNG	0.8382	0.1856/0.3501	102.352	0.4737/0.6512
IL4	0.9480	0.7377/NA	0.2238	0.6298/NA
IL10	0.7887	0.1109/0.2451	41.807	0.0048/0.0192
TGF-β	0.6387	0.0363/0.1084	417.792	0.0320/0.089

Supplemental Table 3 (related to Figure 3). Transcriptional analysis of differential cytokine expression in DC, Mo and CD4 T cells across the different patient populations.

Patient cohort and ID	HLA-B alleles (allele1; allele2)
NT2 444154	08:01 (OR 1.8); 44:03 (OR 1.3)
NT2 811415	07:02 (OR 2.2); 52:01 (OR 0.1)
NT2 339349	44:03 (OR 1.3); 44:03 (OR. 1.3)
NT2 363447	07:02 (OR 2.2); 14:01 (OR 0.8)
NT2 168471	07:02 (OR 2.2); 07:05 (OR nd)
NT2 284579	15:03 (OR 1.2) ;40:06 (OR 0.0)
NT2 187891	14:02 (OR 0.5); 44:02 (OR 0.6)
NT2 674674	13:01 (OR 0.5); 13:02 (OR 0.6)
NT2 176539	14:02 (OR 0.5); 57:01 (OR 0.2)
NT2 787222	44:02 (OR 0.6); 52:01 (OR 0.1)
NT2 838139	35:01 (OR 1.6); 35:03 (OR nd)
NT2 938726	42:01 (OR 1.1); 53:01 (OR 1.3)
NT2 187940	14:03 (OR nd); 15:01 (OR 1.0)
NT2 745577	40:01 (OR 1.7); 44:03 (OR 1.3)
NT2 314532	58:01 (OR 0.6); 58:02 (OR nd)
NT2 237709	07:02 (OR 2.2); 15:01 (OR 1.0)
NT2 967859	42:01 (OR 1.1); 53:01 (OR 1.3)
NT2 937255	53:01 OR 1.3); 58;01 (OR 0.6)
NT2 379234	35:01 (OR 1.6); 52:01 (OR 0.5)
NT2 622800	27:05 (OR 0.3); 57:01 (OR 0.2)
NT2 448020	27:05 (OR 0.3); 57:03 (OR 0.1)
NT1 568125	08:01 (OR 1.8); 57:03 (OR 0.1)
NT1 172583	42:01 (OR 1.1); 57:03 (OR 0.1)
NT1 587483	27:02 (OR nd); 44:02 (OR 1.2)
NT1 533586	51:01 (OR 2.1); 57:03 (OR 0.1)
NT1 389876	40:01 (OR 1.7); 40:02 (OR 0.6)
NT1 779974	15:01 (OR 1.0); 57:03 (OR 0.1)
NT1 588800	38:01 (OR 1.5); 38:01 (OR 1.5)
NT1 254912	13:02 (OR 0.6); 57:01 (OR 0.2)
NT1 694604	35:01 (OR 1.6); 58:01 (OR 0.6)
NT1 595424	14:02 (OR 0.5); 51:01 (OR 1.0)
NT1 497698	35:01 (OR 1.6); 51:01 (OR 1.0)
NT1 504350	07:02 (OR 2.2); 14:02 (OR 0.5)
NT1 829848	18:04 (OR nd); 57:01 (OR 0.2)
NT1 785360	14:02 (OR 0.5); 14:02 (OR 0.5)
NT1 185075	44:02 (OR 1.2); 57:01 (OR 0.2)
NT1 196203	14:03 (OR nd); 57:03 (OR 0.1)
NT1 701998	07:02 (OR 2.2); 27:05 (OR 0.3)
NT1 211774	13:02 (OR 0.6); 57:01 (OR 0.2)
NT1 748754	15:01 (OR 1.0); 51:01 (OR 1.0)
NT1 280008	44:03 (OR 1.3); 58:01 (OR 0.6)
NT1 847041	42:01 (OR 1.1); 45:01 (OR 2.6)
NT1 386576	15:01 (OR 1.0); 51:01 (OR 1.0)
NT1 447160	07:02 (OR 2.2); 14:02 (OR 0.5)
NT1 191551	08:01 (OR 1.8); 35:01 (OR 1.6)

NT1 330183	07:02 (OR 2.2); 53:01 (OR 1.6)
NN 724679	40:01 (OR 1.7); 44:02 (OR 1.2)
NN 473516	08:01 (OR 1.8); 57:01 (OR 0.2)
NN 477889	18:01 (OR 1.4); 51:02 (OR 0.0)
NN 756587	44:02 (OR 1.2); 57:03 (OR 0.1)
NN 842279	15:01 (OR 1.0); 27:05 (OR 0.3)
NN 534694	27:05 (OR 0.3); 57:01 (OR 0.2)
NN 902015	18:01 (OR 1.4); 81:01 (OR 0.2)
NN 553064	15:01 (OR 1.0); 27:05 (OR 0.3)
NN 818703	15:01 (OR 1.0); 45:01 (OR 2.6)
NN 164007	41:02 (OR 1.2); 57:01 (OR 0.2)
NN 380401	14:02 (OR 0.5); 57:01 (OR 0.2)
NN 188482	44:03 (OR 1.3); 49:01 (OR 1.4)
NN 701554	35:01 (OR 1.6); 50:01 (OR 1.0)
NN 929364	35:01 (OR 1.6); 44:03 (OR 1.3)
NN 269198	38:01 (OR 1.5); 57:01 (OR 0.2)

Supplemental Table 4 (related to Figure 1). Detail of HLA-B alleles present in each patients from our NT1, NT2 and NN study cohorts. Those alleles with OR> 1.5 and OR <0.5 were classified as High Risk and Protective alleles, respectively.
